# SMIT (Sodium-Myo-Inositol Transporter) 1 Regulates Arterial Contractility Through the Modulation of Vascular Kv7 Channels

**DOI:** 10.1161/ATVBAHA.120.315096

**Published:** 2020-08-13

**Authors:** Vincenzo Barrese, Jennifer B. Stott, Samuel N. Baldwin, Gema Mondejar-Parreño, Iain A. Greenwood

**Affiliations:** 1 Vascular Research Centre, Institute of Molecular & Clinical Sciences, St George’s, University of London, United Kingdom (V.B., J.B.S., S.N.B., I.A.G.).; 2 Department of Neuroscience, Reproductive Science and Dentistry, University of Naples Federico II, Italy (V.B.).; 3 Department of Pharmacology and Toxicology. School of Medicine, Universidad Complutense de Madrid, Spain (G.M.-P.).

**Keywords:** KCNQ, Kv7, myo-inositol, SLC5A3, SMIT1

## Abstract

**Objective:**

The SMIT1 (sodium:myo-inositol transporter 1) regulates myo-inositol movement into cells and responses to hypertonic stimuli. Alteration of myo-inositol levels has been associated with several diseases, including hypertension, but there is no evidence of a functional role of SMIT1 in the vasculature. Recent evidence showed that in the nervous system SMIT1 interacted and modulated the function of members of the Kv7 family of voltage-gated potassium channels, which are also expressed in the vasculature where they regulate arterial contractility. Therefore, in this study, we evaluated whether SMIT1 was functionally relevant in arterial smooth muscle.

**Approach and Results:**

Immunofluorescence and polymerase chain reaction experiments revealed that SMIT1 was expressed in rat renal and mesenteric vascular smooth muscle cells. Isometric tension recordings showed that incubation of renal arteries with raffinose and myo-inositol (which increases SMIT1 expression) reduced the contractile responses to methoxamine, an effect that was abolished by preincubation with the pan-Kv7 blocker linopirdine and by molecular knockdown of Kv7.4 and Kv7.5. Knockdown of SMIT1 increased the contraction of renal arteries induced by methoxamine, impaired the response to the Kv7.2–Kv7.5 activator ML213 but did not interfere with the relaxant responses induced by openers of other potassium channels. Proximity ligation assay showed that SMIT1 interacted with heteromeric channels formed by Kv7.4 and Kv7.5 proteins in both renal and mesenteric vascular smooth muscle cells. Patch-clamp experiments showed that incubation with raffinose plus myo-inositol increased Kv7 currents in vascular smooth muscle cells.

**Conclusions:**

SMIT1 protein is expressed in vascular smooth muscle cells where it modulates arterial contractility through an association with Kv7.4/Kv7.5 heteromers.

## Abstract 2

Supplemental Digital Content is available in the text.

**Highlights**

- The myo-inositol transporter SMIT1 (sodium:myo-inositol transporter 1) modulates arterial contractility.
- SMIT1 interacts with Kv7.4/Kv7.5 heteromeric channels in the vasculature and affect their function.
- This is the first evidence of the interaction of a transporter with a K^+^ channel in the vasculature.

The cyclic polyol myo-inositol is an organic osmolyte that plays a pivotal role in many cell types, regulating their response to hypertonic environments. Intracellular levels of myo-inositol are tightly controlled by the activity of membrane transporters belonging to the SLC5A (solute carrier family 5A) that mediate the influx of substrates along with sodium. Two SMIT (sodium:myo-inositol transporter) have been cloned and named SMIT1 (SLC5A3)^1^ and SMIT2 (SLC5A11).^2^ Since their first identification in the kidney, myo-inositol transporters have been found in several other tissues including the brain, liver, pancreas, placenta, heart and skeletal muscle.^3,4^ Expression of SMIT1 is upregulated by extracellular hypertonicity via transcriptional mechanisms, resulting in enhanced uptake of myo-inositol by cells. This prevents an increase in the concentration of inorganic ions without perturbing the activity of macromolecules.^5^ Alterations of SMIT1 function have been associated with several diseases, including Down syndrome, Alzheimer disease, and mood disorders.^6^ Moreover, alterations of myo-inositol metabolism have been observed in other diseases, such as epilepsy,^7,8^ stroke,^9^ and diabetes mellitus.^10^ Administration of myo-inositol has been proposed as a potential therapeutic strategy in these conditions.

In addition to its osmotic role, myo-inositol is a substrate for the biosynthesis of phosphatidylinositol, a membrane molecule that is the precursor of phosphatidylinositol-4,5-bisphosphate (PIP_2_), a key modulator of many ion channels.^6,11^ Consequently, myo-inositol transporters may have a profound effect on ion channel activity. Regulation of ion channels function in the vasculature, in particular K^+^ channels, is a key mechanism modulating arterial contractility.

KCNQ-encoded potassium channels (Kv7.1–7.5) are important regulators of cellular activity in neurons, cardiac myocytes, epithelia, and smooth muscle cells, which have an exquisite reliance upon local PIP_2_ levels.^12^ Within the vasculature, Kv7.1, Kv7.4, and Kv7.5 are the main subunit expressed, have been shown to regulate arterial contractility at rest, and contribute to receptor-mediated vasorelaxation.^12,13^ Recently, SMIT1 has been shown to interact with Kv7.1, Kv7.2, and Kv7.3 resulting in increased activity of the channels.^8,14^ These so-called chansporter complexes^15^ have been implicated as key determinants of neuronal activity,^8,16,17^ suggesting a tight link between regulation of osmolarity and cell excitability. Acute exposure to hypertonic solution causes vasodilation in several arterial beds including skeletal muscle arterioles, mesenteric and coronary arteries, through poorly defined mechanisms.^18–20^ Moreover, increased urinary depletion of myo-inositol has been observed in diabetic patients and in animal models of metabolic diseases including hypertension.^21,22^ However, there is little data about the expression of myo-inositol transporters in the arteries,^23^ and there is no evidence about their functional role in vascular smooth muscle cells (VSMCs). We, therefore, set out to establish expression of SMIT1 in VSMCs and ascertaining if modifying SMIT1 levels altered arterial contractility potentially through an interaction with Kv7 channels.

## Materials and Methods

Please see the Major Resources Table in the Data Supplement. Detailed methods are available in the Data Supplement. The authors declare that all supporting data are available within the article (and its Data Supplement files).

### Arteries

Main conduit renal and third-order mesenteric arteries were microdissected and cleaned of adherent fat in ice-cold Krebs solution, as previously described.^24^ Arteries were incubated in DMEM/F-12 culture medium (Sigma Aldrich, Dorset, United Kingdom) supplemented with 1% penicillin-streptomycin (Sigma Aldrich, Dorset, United Kingdom) in a 37°C incubator with 5% CO_2_ for 16 hours (for myo-inositol/raffinose treatments) or 48 hours (for gene-silencing experiments). In double knockdown experiments, arteries were incubated in the morpholinos transfection mix for 32 hours and then incubated in medium containing either vehicle or raffinose plus myo-inositol for 16 hours.

### Cell Culture

Chinese Hamster Ovary (CHO) cells were maintained in DMEM supplemented with 10% (v/v) FBS, 2 mmol/L L-glutamine, and 1% (v/v) penicillin/streptomycin (Sigma Aldrich, Dorset, United Kingdom) in a 37°C incubator with 5% CO_2_. CHO cells were plated in 6-well dish and transfected with pcDNA3.1-KCNQ4, pcDNA3.1-KCNQ5, pcDNA3.1-SMIT1, and pGFP (green fluorescent protein; pMaxCloningTM Vector, Lonza, Basel, Switzerland) plasmids using Lipofectamine 2000 (Thermo Fisher, Paisley, United Kingdom), according to the manufacturer’s instructions. CHO cells were used 24 hours after transfection.

### Isolation of Vascular Smooth Muscle Cells

VSMCs were isolated as previously described.^24^ Arteries were digested with collagenase type IA (2 mg/mL) and protease type X (1 mg/mL). For quantitative polymerase chain reaction (PCR) experiments, VSMCs were prepared to ensure removal of endothelium as previously described.^25^

### Gene Knockdown

Knockdown of Kv7.4, Kv7.5, and SMIT1 in renal and mesenteric arteries was performed by transfection with morpholino oligonucleotides as described in vessels previously.^26^ Targeting and scrambled (control) morpholino oligonucleotides (5 µmol/L; Genetools, Oregon) were mixed in Opti-MEM and transfected using Lipofectamine 2000 (Thermo Fisher, Paisley, United Kingdom).

### RNA Extraction, Reverse Transcription and Quantitative PCR

Total RNA was extracted from arteries using Monarch Total RNA Miniprep Kit (New England Biolabs, Hitchin, United Kingdom), according to the manufacturer’s instruction, and reverse transcribed to cDNA using Luna Script RT Super Mix (New England Biolabs, Hitchin, United Kingdom). Quantitative PCR experiments were run in CFX96 Real-Time PCR Detection System (Bio-Rad, Hertfordshire, United Kingdom) using the SYBR-Green detection technique and specific primers (Table I in the Data Supplement), as previously described.^24^

### Immunofluorescence

VSMCs were fixed with 3% paraformaldehyde, treated with 0.1 mol/L glycine, and incubated with primary antibodies overnight at 4°C, as previously described.^16^ Coverslips were then washed with PBS and incubated for 1 hour with secondary antibodies conjugated to Alexa Fluor 488 or Alexa Fluor 567. Coverslips were analyzed with a Nikon A1R confocal microscope (Nikon Instruments Europe BV, Amsterdam, Netherlands). Corrected total cell fluorescence was calculated using ImageJ software as elsewhere described.^27^ The number of cells analyzed is indicated by n, whereas N represents the number of animals used.

### Patch-Clamp Recordings

All current recordings were made with an Axopatch 200B and a Digidata 1322A (Axon Instruments, Burlingame, CA). For renal VSMCs, membrane Kv currents were measured using perforated whole-cell voltage-clamp by including amphotericin B in the pipette solution (final concentration of 200 μg/mL) at room temperature (21°C–23°C).

### Wire Myography

Segments of renal arteries (≈2 mm) were mounted in a wire myograph (Danish Myo Technology, Aarhus, Denmark) for isometric tension recording.^28^ Concentration-effect curves for methoxamine (30 nmol/L–30 µmol/L) or KCl (10–120 mmol/L) were constructed to evaluate arterial contractility after the experimental treatments. Data were recorded and analyzed using LabChart 7 (ADInstruments, Dunedin, New Zealand).

### Proximity Ligation Assay

Proximity ligation assay (PLA) was used to assess protein-protein interactions as previously described using validated antibodies.^24,26^ Analysis of midcell xy sections was performed using ImageJ software. For PLA in CHO cells, the number of puncta per cell was calculated by dividing the total number of dots by the number of nuclei in the microscopic field.

### Drugs

Specific activators of different potassium channels were used at submaximal concentrations derived from previous work. These were the Kv7.2–7.5 activators ML213 and retigabine^29^ (Tocris Bioscience, Bristol, United Kingdom), the Kv7.1 activator RL-3^30^ (Sigma Aldrich, Dorset, United Kingdom), the K_ATP_ activator levcromakalim^31^ (Tocris Bioscience, Bristol, United Kingdom), and the BK_Ca_ activator NS11021^32^ (Tocris Bioscience, Bristol, United Kingdom). Specific blockers of Kv7 channels (XE991,^24^ linopirdine and HMR1556^33^), SMIT1 (phlorizin^8^), and the G-protein-gated inwardly rectifying K^+^ channels (Tertiapin Q^34^) were purchased from Tocris Bioscience, Bristol, United Kingdom.

### Statistical Analysis

All data are expressed as mean±SEM. One- or 2-way ANOVA test followed by a Bonferroni, Dunnett, or Tukey multiple comparisons test, and Student *t* test (paired or unpaired) were used to determine statistical significance between groups, according to the different experiments. The significance level for statistic tests was 0.05 (differences were considered statistically significant when *P*<0.05).

## Results

### SMIT1 Is Expressed in Rat VSMCs

**Figure 1.**
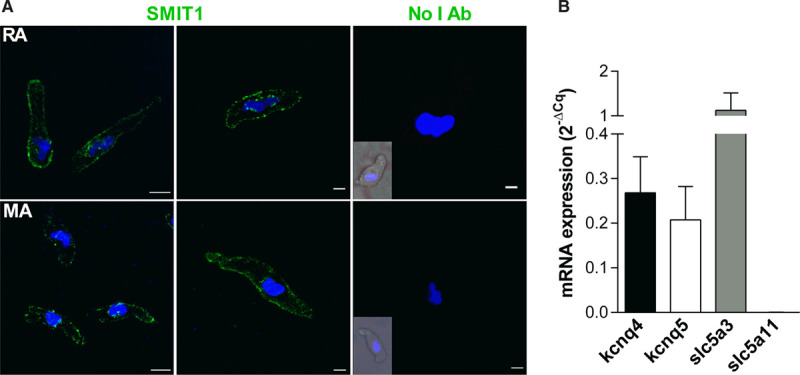
**Expression of SMIT1 (sodium:myo-inositol transporter 1) in vascular smooth muscle cells (VSMCs).** **A**, Representative immunofluorescence showing the expression of SMIT1 protein in VSMCs isolated from renal arteries (RA, **top**) and mesenteric arteries (MA, **bottom**). The **right**-most parts show the staining in absence of the SMIT1 antibody (No I Ab). The insets show a brightfield image of the cell. Nuclei (DAPI [4',6-diamidino-2-phenylindole, dihydrochloride] staining, blue) are also shown. Scale bar=10 µm (**left**-most parts) and 5 µm (other parts). **B**, Quantitative polymerase chain reaction showing the expression of the transcripts encoding for Kv7.4 (kcnq4), Kv7.5 (kcnq5), SMIT1 (slc5a [solute carrier family 5A] 3) and SMIT2 (slc5a11) in VSMCs isolated from renal arteries. Data are expressed using the 2^-ΔCq^ formula (N=5).

Immunofluorescence experiments on freshly dispersed arterial VSMCs isolated from rat renal and mesenteric arteries were performed to determine whether SMIT1 was present in vascular smooth muscle cells. Images in Figure 1A show that SMIT1 was expressed in renal and mesenteric VSMCs, with a predominantly peripheral, membrane-like distribution. Quantitative PCR in purified renal VSMCs showed an abundant expression of SMIT1 (slc5a3), whereas levels of SMIT2 (slc5a11) were negligible (Figure 1B).

### Modulation of SMIT1 Levels Regulates Arterial Contractility

**Figure 2.**
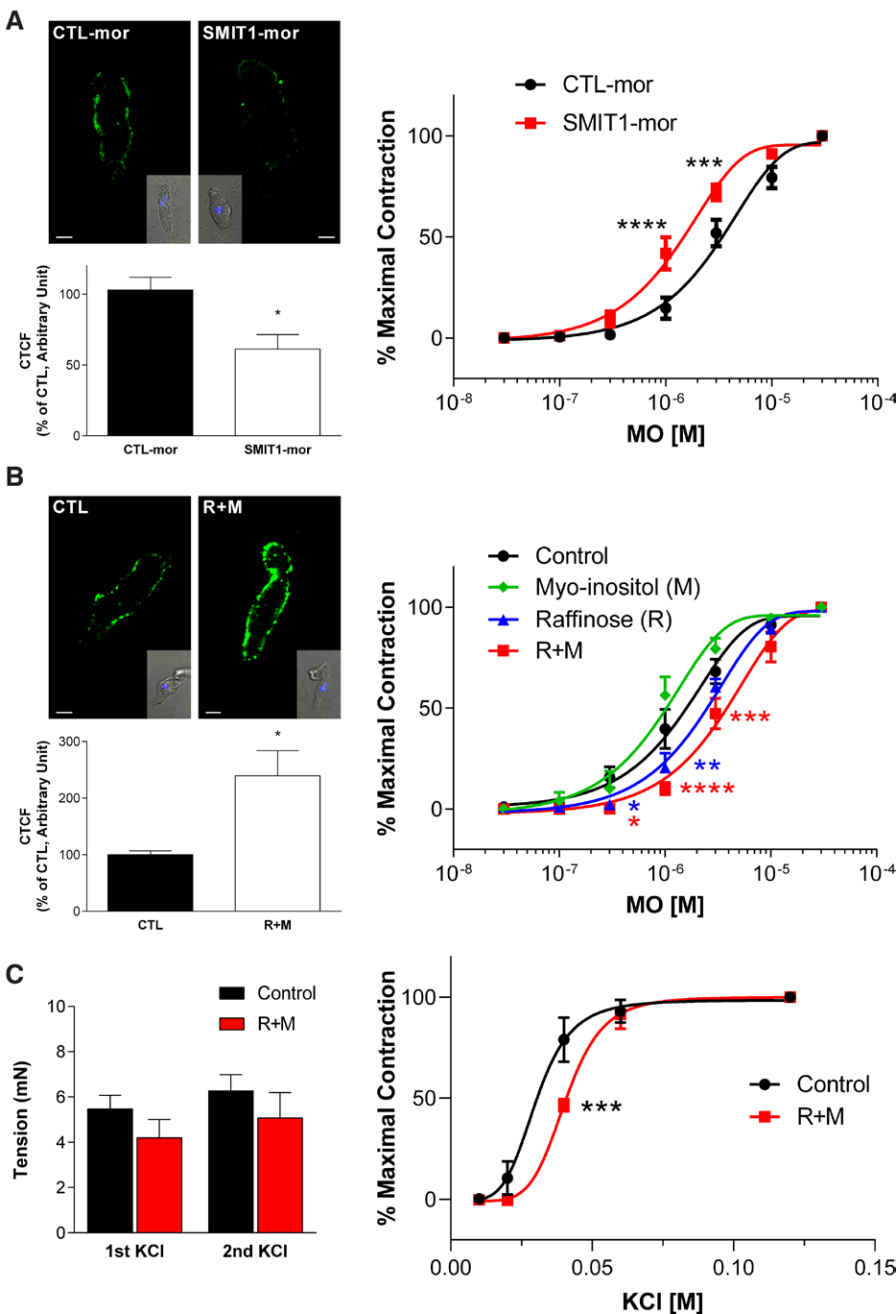
**Effects of the modulation of SMIT1 (sodium:myo-inositol transporter 1) levels on arterial contractility.** **A**, **left**, representative images showing the immunostaining of SMIT1 in renal vascular smooth muscle cells (VSMCs) incubated with a scrambled (CTL-mor) or an anti-SMIT1 morpholino oligonucleotide (SMIT1-mor). The insets show brightfield images of the cells. Scale bar=5 µm. The graph shows the quantification of SMIT1 fluorescence intensities measured as corrected total cell fluorescence (CTCF) and expressed as percentage of control (CTL-mor). Data are expressed as mean±SEM. *P<0.05 vs respective controls; n=28 cells for CTL-mor and 30 cells for SMIT1-mor obtained from 3 different preparations (N=3). **Right**, isometric tension recordings showing the effects of increasing concentration of methoxamine (MO) in segments of renal arteries after 48 h incubation with CTL-mor (black line) or SMIT1-mor (red line). Data are shown as percentage of the contraction obtained with 30 µmol/L MO. Data are expressed as mean±SEM. ***P<0.001; ****P<0.0001 vs controls; N=13 for CTL-mor and 16 for SMIT1-mor. **B**, **left**, representative images showing the immunostaining of SMIT1 in renal VSMCs incubated with vehicle (CTL) or raffinose plus myo-inositol (R+M). The insets show brightfield images of the cells. Scale bar=5 µm. The graph shows the quantification of SMIT1 fluorescence intensities measured as CTCF and expressed as percentage of control. Data are expressed as mean±SEM. *P<0.05 vs respective controls; n=28 cells for CTL and 30 cells for R+M from 3 different preparations (N=3) per experimental group. **Right**, isometric tension recordings showing the effects of increasing concentration of MO in segments of renal arteries after 16 h incubation with vehicle (control, black line, N=10), 1 mmol/L myo-inositol (M, green line, N=6), 150 mmol/L raffinose (R, blue line; N=8), or 150 mmol/L raffinose plus 1 mmol/L myo-inositol (R+M, red line, N=10). Data are shown as percentage of the contraction obtained with 30 µmol/L MO. Data are expressed as mean±SEM. *P<0.05; **P<0.01; ***P<0.001; ****P<0.0001 vs controls. **C**, **left**, isometric tension recordings showing the effects of 60 mmol/L KCl in renal arteries after 16 h incubation with vehicle (control, black bars, N=20), or 150 mmol/L raffinose plus 1 mmol/L myo-inositol (R+M, red bars, N=22). Arteries were challenged with 2 independent KCl stimuli. **Right**, Isometric tension recordings showing the effects of increasing concentration of KCl in segments of renal arteries after 16 h incubation with vehicle (control, black line) or 150 mmol/L raffinose plus 1 mmol/L myo-inositol (R+M, red line). Data are shown as percentage of the contraction obtained with 120 mmol/L KCl. Data are expressed as mean±SEM. ***P<0.001; ****P<0.0001 vs controls; N=6 per experimental group.

Renal arteries were incubated overnight with different treatments known to potentiate or reduce SMIT1 function and expression,^17^ and vascular reactivity was evaluated by measuring contractions to increasing concentration of the α1-adrenergic receptors agonist methoxamine (30 nmol/L–30 µmol/L). Incubation of renal arteries with a SMIT1-specific morpholino oligonucleotide reduced SMIT1 expression by ≈40% (Figure 2A, left) and produced a left-shift in the concentration-response curve for methoxamine with respect to control (at 1 µmol/L methoxamine: 40±8% of maximal contraction with SMIT1-morpholino; 14±5% for scrambled control; Figure 2A, right). Neither 3-hour nor 16-hour (overnight) incubation with the SMIT1 transport inhibitor phlorizin (500 µmol/L) modified methoxamine-induced contraction of renal artery (Figure I in the Data Supplement).

Previous studies showed that SMIT1 abundance in neuronal cell membranes is increased by bathing solutions made hypertonic by addition of raffinose.^17,23^ Overnight incubation of renal arteries with 150 mmol/L raffinose plus the transporter substrate myo-inositol (1 mmol/L) increased SMIT1 fluorescence intensity in VSMCs by ≈140% with respect to controls (Figure 2B, left) and reduced concentration-dependent contractions to methoxamine (at 1 µmol/L methoxamine: 10±3% of maximal contraction with raffinose plus myo-inositol; 39±10% for control; Figure 2B). Incubation with raffinose alone also inhibited contractions to methoxamine, although to a lesser extent as raffinose plus myo-inositol (at 1 µmol/L methoxamine: 20±7% of maximal contraction; Figure 2B). In contrast, overnight incubation with myo-inositol alone did not affect renal artery contractility. Addition of the SMIT1 transport inhibitor phlorizin (500 µmol/L) had a small effect on the anticontractile action of raffinose plus inositol (Figure IIA in the Data Supplement). Moreover, wortmannin (10 nmol/L), that depletes PIP_2_ levels, partially prevented the right-shift of the concentration-response curve to methoxamine induced by raffinose plus myo-inositol, being effective only at 3 µmol/L methoxamine (82±4% of maximal contraction in wortmannin and raffinose plus myo-inositol; 54±12% in raffinose+myo-inositol; Figure IIB in the Data Supplement).

Incubation or renal arteries with raffinose plus myo-inositol did not modify the contractile response induced by 60 mmol/L KCl (Figure 2C, left). When exposed to increasing concentration of KCl (10–120 mmol/L), incubation of renal arteries with raffinose plus myo-inositol caused a reduction of the contraction only at lower KCl concentration with respect to controls (at 40 mmol/L KCl: 46±7 of maximal contraction with raffinose plus myo-inositol; 80±11% in control), a result consistent with the involvement of K^+^ channels in mediating the effects on arterial contractility induced by raffinose plus myo-inositol (Figure 2C, right).

Overall, these results show that augmenting membrane expression of SMIT1 attenuated vasoconstrictor responses with a mechanism that was partly dependent on increased intracellular availability of myo-inositol.

### SMIT1 Selectively Modulates the Functional Impact of Specific Kv7 Subunits

**Figure 3.**
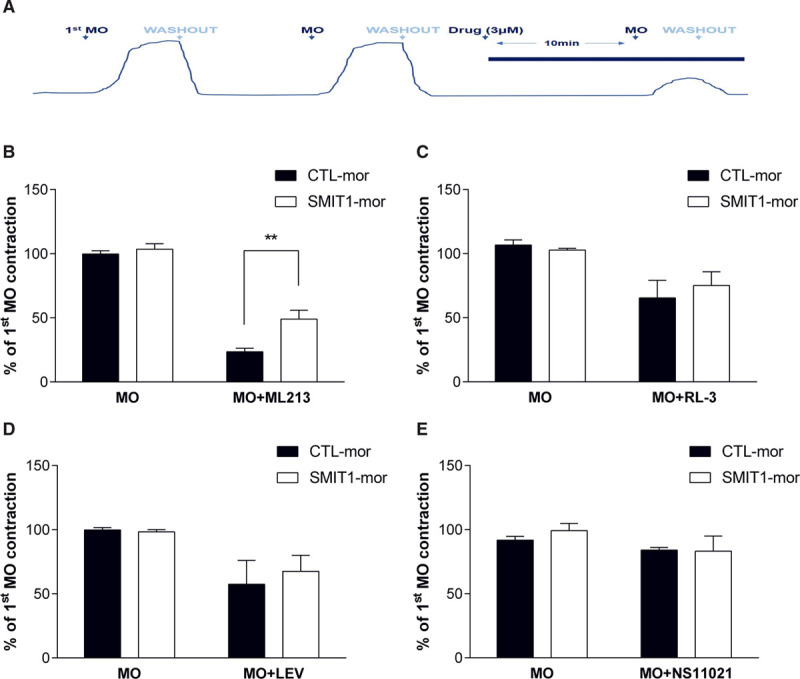
**Effects of SMIT1 (sodium:myo-inositol transporter 1)-silencing on K+ channels activators.** **A**, Representative image showing the protocol used for isometric tension recordings. After 2 stimulations with 10 µmol/L methoxamine (MO), segments of renal arteries were incubated for 10 min with K^+^ channels activators (all used at a concentration of 3 µmol/L) and then stimulated again with MO. **B–E**, Quantification of the responses to MO in the presence of ML213 (**B**, N=9), RL-3 (**C**, N=6), levcromakalim (**D**, N=5), or NS11021 (**E**, N=4) in renal arteries incubated with a scrambled (CTL-mor) or an anti-SMIT1 morpholino oligonucleotide (SMIT1-mor). Data are expressed as mean±SEM. and shown as percentage of the contraction to the first stimulation with MO (see **A**). The responses to the second incubation of MO before the incubation with the activators (MO) are also shown. **P<0.01.

**Figure 4.**
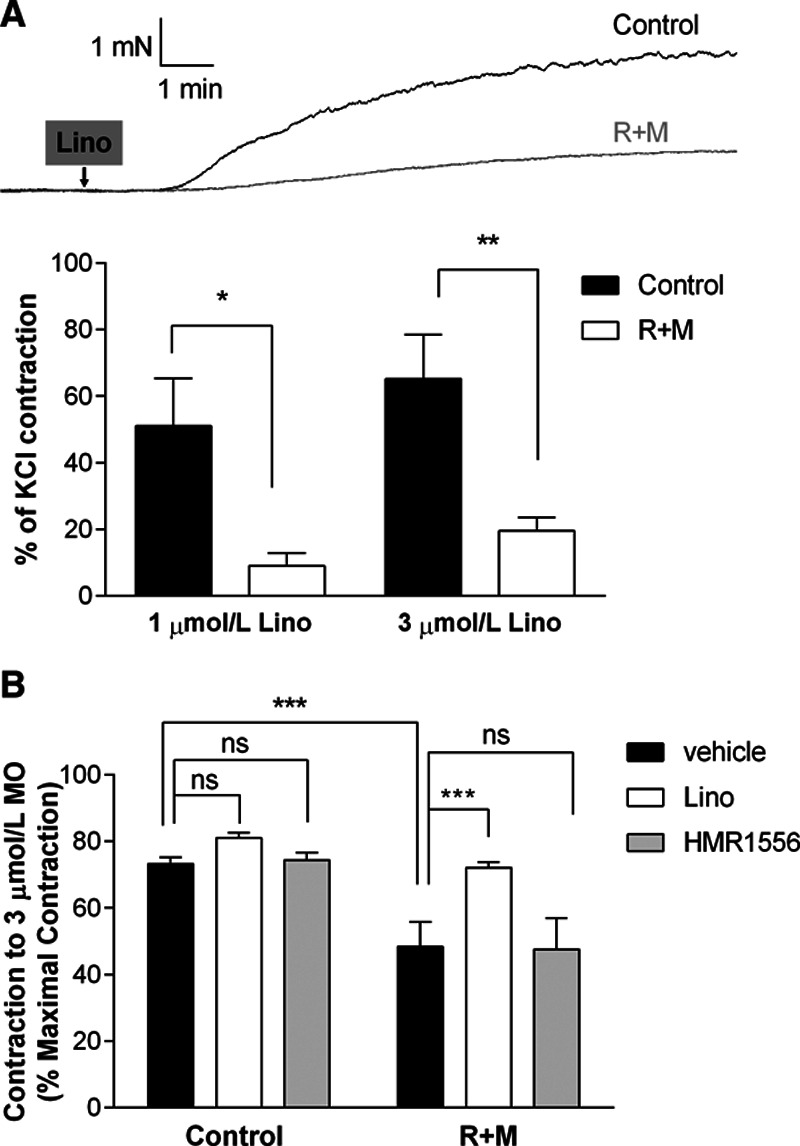
**Effects of enhanced SMIT1 (sodium:myo-inositol transporter 1) expression on Kv7 channels blockers.** **A**, **top**, representative traces showing the effects on isometric tension recordings of the pan-Kv7 blocker linopirdine (3 µmol/L) in renal arteries after 16 h incubation with vehicle (black line) or raffinose plus myo-inositol (R+M; gray line). **Bottom**, bar graphs showing the contraction to linopirdine (1 and 3 µmol/L) of renal arteries incubated for 16 h with vehicle (control, black bars) or R+M (white bars) in wire-myography experiments. Data are expressed as mean±SEM and shown as percentage of the initial contraction to 60 mmol/L KCl. *P<0.05, **P<0.01; N=10 per experimental group. **B**, bar graphs showing the effects of methoxamine (3 µmol/L) in the presence of dimethyl sulfoxide (DMSO; vehicle, black bars), 3 µmol/L linopirdine (Lino, white bars) or 1 µmol/L HMR1556 (gray bars) on isometric tension recordings of renal arteries incubated for 16 h with vehicle (control) or R+M. Data are expressed as mean±SEM and shown as percentage of the contraction to 30 µmol/L methoxamine. ***P<0.001; ns=not significant; N=10 per experimental group.

Having established that SMIT1 modulates arterial function, we investigated whether these effects occurred through the regulation of the activity of K^+^ channels, which are key regulators of arterial contractility. To this aim, we evaluated whether modulating SMIT1 levels interfered with the ability of several activators of different K^+^ channel classes found in the vasculature to counteract the vasoconstriction of renal artery segments induced by 10 µmol/L methoxamine (Figure 3A). Under control conditions methoxamine-induced contractions were reduced by preincubation with the Kv7.2–Kv7.5 activator ML213 (3 µmol/L; 24±3% of first contraction to methoxamine), the Kv7.1-specific activator RL-3 (3 µmol/L; 65±13%), the K_ATP_- opener levcromakalim (3 µmol/L; 57±18%), and the BK_Ca_ activator NS11021 (3 µmol/L; 84±2%; Figure 3B through 3E, black bars). Knockdown of SMIT1 caused a significant impairment of the ML213 effect on methoxamine-induced contraction (49±7% of first contraction to methoxamine, Figure 3B, white bars), whereas no changes were observed in the responses to the other K^+^ channels activators (RL-3: 75±10%; LEV: 67±12%; NS11021: 83±11%; Figure 3C through 3E, white bars). In contrast to the knockdown studies, increasing SMIT1 levels by incubation with raffinose and myo-inositol did not alter the vasorelaxant properties of any of the selected K^+^ channels openers when compared with their respective controls (Figure III in the Data Supplement). To further investigate the possible involvement of Kv7 channels in the anticontractile effect of SMIT1, we performed myography experiments with the pan-Kv7 blocker linopirdine. Application of linopirdine (1–3 µmol/L) contracted renal arteries considerably less in segments incubated with raffinose and myo-inositol (Figure 4A) and abrogated the reduced contraction to methoxamine produced by treatment with raffinose and myo-inositol (Figure 4B). Similar effects were not seen with the selective Kv7.1 blocker HMR1556 (1 µmol/L; Figure 4B) nor tertiapin Q (1 µmol/L), a blocker of the inward-rectifier K^+^ channel GIRK (Figure IV in the Data Supplement).

**Figure 5.**
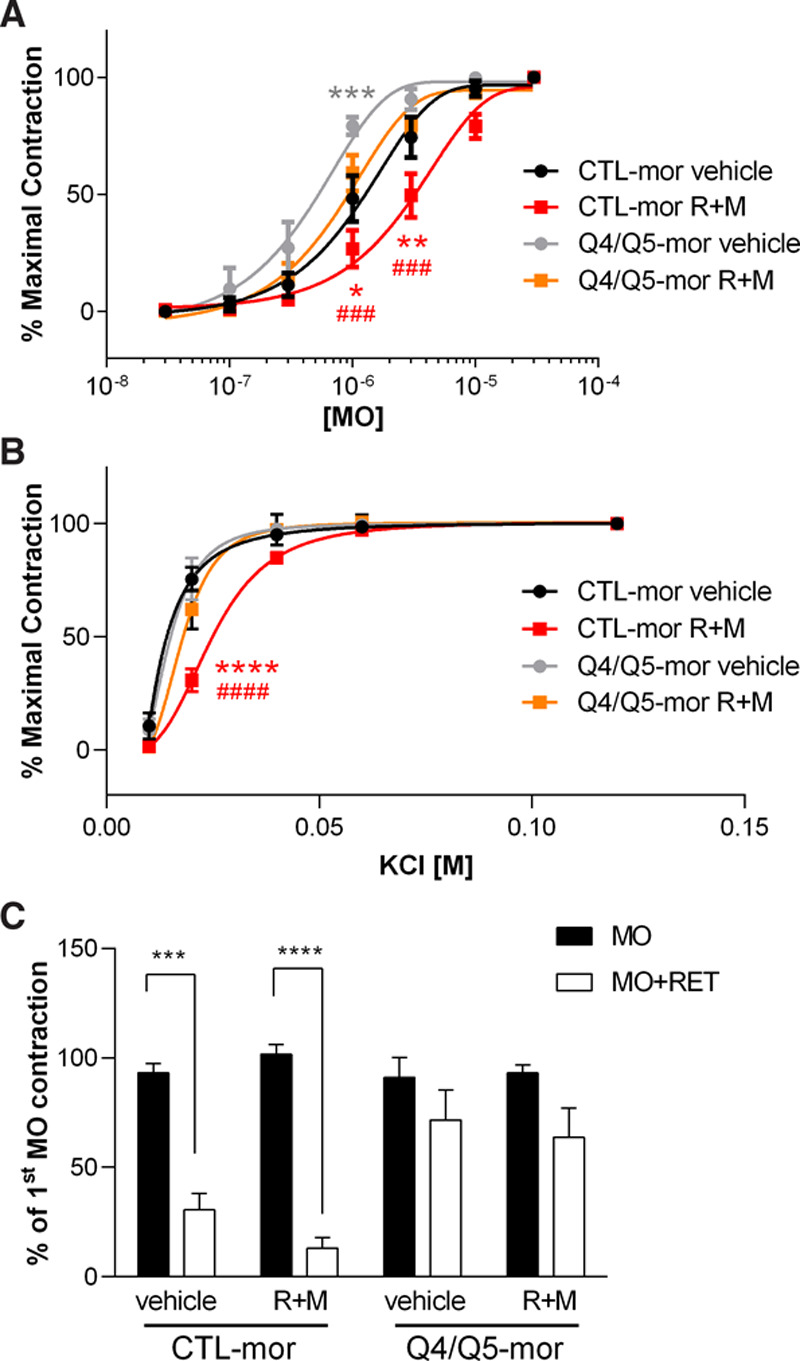
**Effects of enhanced SMIT1 (sodium:myo-inositol transporter 1) expression and silencing of Kv7.4 and Kv7.5 in renal arteries.** Isometric tension recordings showing the effects of increasing concentration of methoxamine (MO, **A**) or KCl (**B**) in segments of renal arteries transfected with a scrambled morpholino (CTL-mor) or 2 morpholinos targeting Kv7.4 and Kv7.5 (Q4/Q5-mor). 32 h after the transfection, arteries were incubated for 16 h with either vehicle (black and gray lines) or 150 mmol/L raffinose plus 1 mmol/L myo-inositol (raffinose plus myo-inositol [R+M]; red and orange lines). Data are shown as percentage of the contraction obtained with 30 µmol/L MO (**A**) or 120 mmol/L KCl (**B**). Data are expressed as mean±SEM. *P<0.05; **P<0.01: ****P<0.0001 vs CTL-mor vehicle; ###*P*<0.001; ####*P*<0.0001 vs Q4/Q5-mor R+M; N=6 for vehicle incubated arteries and 8 for Q4/Q5-mor arteries. **C**, Bar graph showing the effects of the Kv7 activator retigabine (RET) in Kv7.4 and Kv7.5-silenced arteries. At the end of the experiments shown in **A** and **B**, arteries were challenged twice with 10 µmol/L MO alone (second MO contraction is shown with black bars) and then with MO in the presence of 10 µmol/L RET (white bars). Data are expressed as mean±SEM and shown as percentage of the contraction to the first stimulation with MO. ****P<0.0001; N=6 for vehicle incubated arteries and 8 for Q4/Q5-mor arteries.

To further investigate the involvement of Kv7.4 and Kv7.5 channels in mediating the effects of SMIT1 on arterial contractility, we reduced Kv7.4 and Kv7.5 channels levels with morpholino oligonucleotides (Q4/Q5-mor). Knockdown of Kv7.4 and Kv7.5 prevented the rightward-shift of the concentration-response curves for methoxamine and KCl (Figure 5A and 5B) induced by incubation with raffinose plus myo-inositol. The effective knockdown of Kv7.4 and Kv7.5 channels was confirmed by the lack of vasorelaxant effects of the Kv7 activator retigabine (10 µmol/L) in arteries transfected with morpholinos targeting Kv7.4 and Kv7.5 (Figure 5C). Overall, these data show that SMIT1 influences vascular contractility via a mechanism dependent, in part, on Kv7.4/Kv7.5.

### SMIT1 Interacts With Heteromeric Kv7.4–Kv7.5 Channels

**Figure 6.**
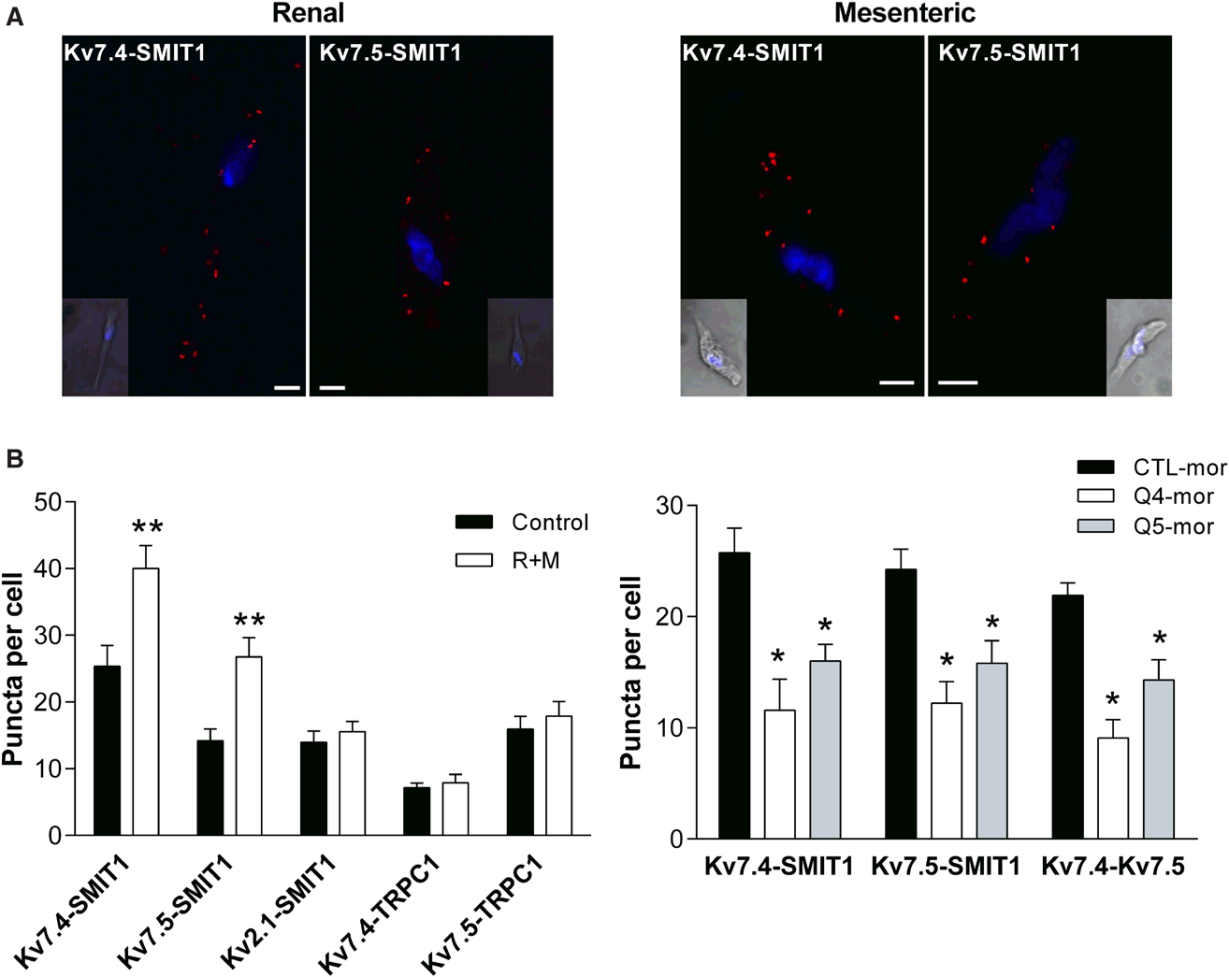
**Interaction of SMIT1 (sodium:myo-inositol transporter 1) with vascular Kv7 channels.** **A**, Proximity Ligation Assays (PLAs) showing the interaction of SMIT1 with Kv7.4 and Kv7.5 in vascular smooth muscle cells (VSMCs) isolated from renal and mesenteric arteries. The insets show a brightfield image of the cell. Nuclei (DAPI [4',6-diamidino-2-phenylindole, dihydrochloride] staining, blue) are also shown. Scale bar=5 µm. **B**, Bar graphs showing the interactions of Kv7.4, Kv7.5, and SMIT1 with Kv2.1 and TRPC1 (transient receptor potential C1 channel) in VSMCs isolated from renal arteries incubated for 16 h with vehicle (control, black bars) or 150 mmol/L raffinose plus 1 mmol/L myo-inositol (raffinose plus myo-inositol [R+M], white bars). Data represent the mean number of PLA signals per midcell xy section, expressed as mean±SEM. n=26–36 cells from 3 to 4 rats (N=3–4) per experimental point in 3–4 sessions. *P<0.05 vs respective controls. **C**, Bar graphs showing the interaction between Kv7.4-SMIT1, Kv7.5-SMIT1, and Kv7.4–Kv7.5 in renal VSMCs. Arteries were incubated with a scrambled morpholino (CTL-mor, black bars), a morpholino targeting Kv7.4 (Q4-mor, white bars), or a morpholino against Kv7.5 (Q5-mor, gray bars). Data represent the mean number of PLA signals per midcell xy section, expressed as mean±SEM. n=26–36 cells from 3 to 4 rats (N=3–4) per experimental point in 3 to 4 sessions. *P<0.05 vs respective controls (CTL-mor).

Our myography data suggests that SMIT1 modulates vascular reactivity through altering Kv7 channel activity. The impairment of the ML213-dependent relaxation in SMIT1-silenced arteries and the lack of effect of the Kv7.1-selective drugs (RL-3, HMR1556), along with the impairment of raffinose plus myo-inositol effects in Kv7.4/Kv7.5–silenced arteries suggest the involvement of Kv7.4 and Kv7.5 subunits. Therefore, we performed PLA to investigate whether SMIT1 interacted with Kv7 channel subunits. PLA puncta were detected in VSMCs from renal and mesenteric arteries for both Kv7.4-SMIT1 and Kv7.5-SMIT1 couples (Figure 6A). Interestingly, SMIT1 also interacted with the ancillary KCNE4 subunit that is abundant in VSMCs and colocalizes with Kv7.4/Kv7.5 channels^35^ (Figure VA in the Data Supplement). To get insight into the dynamic changes of these interactions when SMIT1 expression was upregulated, we evaluated the PLA puncta for Kv7.4-SMIT1 and Kv7.5-SMIT1 in renal arteries incubated with raffinose plus myo-inositol. Enhancing SMIT1 expression increased the number of PLA puncta for Kv7.4-SMIT1 and Kv7.5-SMIT1 by ≈60 and ≈85%, respectively. Treatment with raffinose plus myo-inositol did not modify the interaction of SMIT1 with the voltage-gated K^+^ channel Kv2.1 or the interaction of Kv7.4 or Kv7.5 with the TRPC1 (transient receptor potential C1 channel; Figure 6B), suggesting a preferential interaction between SMIT1 and Kv7.4/Kv7.5 channels in these experimental settings.

We then performed further experiments to evaluate the molecular determinant of the interaction between SMIT1 and Kv7 channels by analyzing the PLA puncta for Kv7.4-SMIT1 and Kv7.5-SMIT1 when Kv7 subunits were knocked down with morpholino oligonucleotides. As shown in Figure 6C, morpholino targeting Kv7.4 (Q4-mor) reduced the number of interactions of SMIT1 with Kv7.4 (≈60%) and Kv7.5 (≈50%) in renal arteries. Similarly, a morpholino targeted to Kv7.5 (Q5-mor) reduced SMIT1-Kv7.4 and SMIT1-Kv7.5 interactions by ≈50%. Both morpholinos reduced the number of PLA punctae observed when antibodies for Kv7.4 and Kv7.5 were used with respect to control condition (Figure 6C). Similar results were observed in VSMCs isolated from mesenteric arteries (Figure VB in the Data Supplement). To get further insight into the stoichiometry of these interactions, we performed PLAs on CHO cells overexpressing homomeric Kv7.4 or Kv7.5 channels and heteromeric channels formed by Kv7.4 and Kv7.5. Although PLA signals were detected for Kv7.5-SMIT1 in CHO cells transfected with Kv7.5 only, the number of Kv7.4-SMIT1 interactions in CHO transfected with Kv7.4 was not significantly different from background signals. Interestingly, a significant increase in PLA puncta for Kv7.4 and SMIT1 was detected when Kv7.4 and Kv7.5 were coexpressed in CHO cells (Figure VC in the Data Supplement). These results suggest that SMIT1 interacts with heteromeric channels formed by Kv7.4 and Kv7.5.

### SMIT1 Effects on Kv7-Mediated Currents

**Figure 7.**
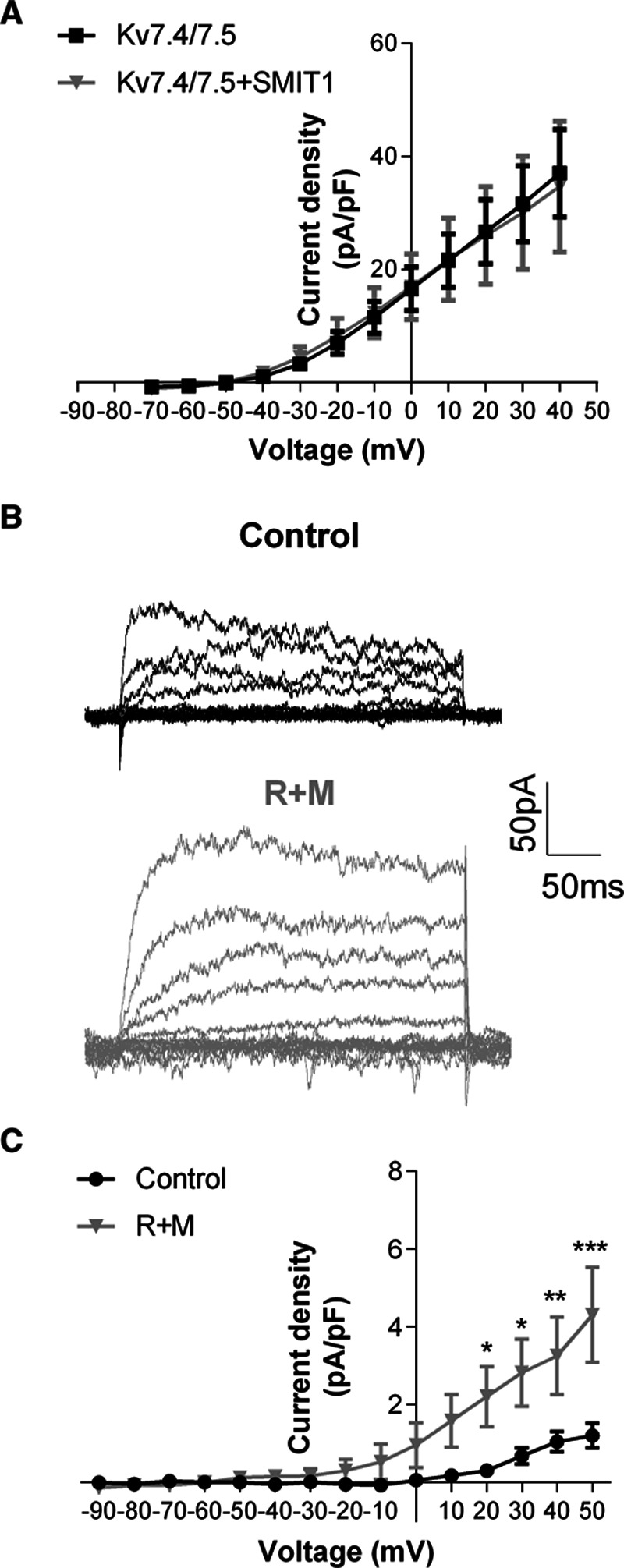
**Effects of SMIT1 (sodium:myo-inositol transporter 1) on Kv7.4/Kv7.5-mediated currents.** **A**, Mean current-voltage relationship in Chinese Hamster Ovary cells transfected with plasmids encoding for Kv7.4 and Kv7.5 plus a GFP (green fluorescent protein)-encoding plasmid (black line) or a plasmid encoding for SMIT1 (gray line). Data are expressed as mean±SEM. n=8 per experimental group. **B**, representative traces showing XE-991 sensitive currents in vascular smooth muscle cells (VSMCs) isolated from renal arteries incubated for 16 h with vehicle (control, black traces) or 150 mmol/L raffinose plus 1 mmol/L myo-inositol (raffinose plus myo-inositol [R+M], gray traces). **C**, Mean current-voltage relationship of the XE991-sensitive current in VSMCs isolated from renal arteries incubated for 16 h with vehicle (control, black line) or 150 mmol/L raffinose plus 1 mmol/L myo-inositol (R+M, gray line). Data are expressed as mean±SEM. n=7 cells for control and 8 cells for R+M, obtained from 8 animals in 8 experimental sessions. *P<0.05; **P<0.01; ***P<0.001 vs control.

In studies on CHO cells, the amplitude of currents produced by the overexpression of Kv7.4 and Kv7.5 was not altered by coexpression with SMIT1 (Figure 7A), but there was a small but nonsignificant effect on the voltage-dependence of activation (V_1/2_=−10.6±4.0 mV for Kv7.4/7.5; V_1/2_=−16.1±3.4 for Kv7.4/7.5 plus SMIT1; n=8 per group; *P*=0.32). In studies on native K^+^ currents in isolated renal artery smooth muscle cells, increasing SMIT1 expression by overnight incubation of the artery in raffinose plus inositol increased XE991-sensitive currents compared with control (Figure 7B and 7C). Raffinose plus inositol had no effect on the membrane capacitance (raffinose plus myo-inositol: 33±1.4 pF; control: 36.0±2.8 pF).

These results show that in identical recording conditions augmenting SMIT1 increased Kv7 currents in VSMCs.

## Discussion

### SMIT1 Regulates Arterial Contractility

In this study, we identified that SMIT1 mRNA and protein are expressed in VSMCs, and altering SMIT1 levels regulated arterial contractility mainly through the modulation of Kv7 channels. Introduction of a morpholino targeting SMIT1 reduced SMIT1 abundance and enhanced vasoconstrictor responses, whereas exposure to hypertonic medium that increased SMIT1 expression reduced arterial contractility. These data provide the first evidence that SMIT1 protein is expressed in the vasculature and has a functional role in the regulation of arterial contractility.

Our results show that methoxamine in raffinose-incubated arteries was less effective compared to arteries incubated in isotonic conditions. These effects were not due to a direct effect of the hypertonicity produced by raffinose because acute application had no relaxant effect and the inhibitory effect was maintained after bathing in isotonic medium for over an hour. We speculated this might be due to increased SMIT1 activity as raffinose (and hyperosmolarity in general) induces the activity of the transcription factor TonEBP (tonicity responsive enhancers binding protein) that increases the expression of SMIT1.^5^ Consistent with this hypothesis, we observed greater membrane abundance of SMIT1 in renal artery smooth muscle cells following incubation with raffinose plus myo-inositol similar to previous reports in neurons.^17^ Further support for a direct role of SMIT1 in the regulation of vascular contractility was provided by the observation that SMIT1-silenced arteries were significantly more contractile than control arteries. Consequently, there is a correlation between changes in SMIT1 abundance and contractile responses to methoxamine concurrent with SMIT1 regulating arterial contractility. These effects do not appear to be mediated primarily by increased availability of intracellular myo-inositol as administration of myo-inositol alone did not mimic the effect of raffinose, and blockade of SMIT1 transport with phlorizin did not prevent the effects on arterial contractility.

### SMIT1 Induces Vasorelaxation Through the Activation of Heteromeric Kv7.4–Kv7.5 Channels

Raffinose plus inositol inhibited contractions produced by lower concentration of K^+^ (≤40 mmol/L) but not higher concentrations consistent with an effect involving K^+^ channels. Opening of K^+^ channels hyperpolarizes the cell membrane potential thus reducing the activation of voltage-gated calcium channels and the consequent influx of calcium ions that is necessary for VSMCs contraction. VSMCs express a wide range of K^+^ channels that regulate arterial contractility,^36,37^ but considerable evidence have been accumulated for a key role of Kv7.4/7.5 channels in the regulation of basal arterial diameter and also the active response to receptor agonists.^38^ Thus, the pan-Kv7 blockers linopirdine and XE991 contract most arteries, whereas Kv7.1-specific blockers like HMR1556 do not show a clear effect on vascular tone.^12^ Impairment of Kv7.4 function and expression have been observed in 2 animal models of hypertension such as spontaneously hypertensive rats and angiotensin II–infused mice,^33,39,40^ and trafficking of Kv7.4 subunits to the plasma membrane is altered upon stimulation with angiotensin II.^24^ Reduction of Kv7 function has also been observed in coronary arteries exposed to high glucose.^41^ Evidence from functional and molecular studies have highlighted that Kv7.4 and Kv7.5 form a heteromeric channel that is the main Kv7 species in VSMCs,^42,43^ with the participation of the accessory subunit KCNE4.^35^ In the present study, knockdown of SMIT1 attenuated the anticontractile effect of the Kv7.2–Kv7.5 specific activator ML213 but did not alter the effectiveness of activators of Kv7.1 (RL-3), BK_Ca_ (NS11021) or K_ATP_ (levcromakalim). The anticontractile effect of increasing SMIT1 levels was prevented by linopirdine but not the Kv7.1 specific blocker HMR1556 nor tertiapin Q, a blocker of PIP_2_-sensitive GIRK channels. A role for Kv7.4 and 7.5 was consolidated by the use of morpholinos targeting Kv7.4 and Kv7.5 that abrogated the inhibitory effect of raising raffinose plus myo-inositol. Interestingly, the effects of ML213 were not potentiated after raffinose and myo-inositol incubation. A possible explanation is that overexpression of SMIT1 heightened the activity of Kv7.4/Kv7.5 sufficiently to negate the stimulatory effect of Kv7 activators. The fact that linopirdine was less able to contract resting arteries incubated with raffinose and myo-inositol supports this hypothesis, although it is possible that other mechanisms involved in arterial contraction might be affected by the increased expression of SMIT1.

A crucial role for Kv7 channels was corroborated by patch-clamp experiments showing that incubation with raffinose and myo-inositol increased the amplitude of Kv7 currents (XE991-sensitive component) in renal VSMCs. Interestingly, when we recorded the effects of SMIT1 on Kv7.4/Kv7.5 currents in heterologous systems, we observed only a trend to increase voltage-sensitivity of the Kv7.4/Kv7.5 channels that was quantitatively similar to that induced by SMIT1 on Kv7.2/Kv7.3 heteromeric channels (≈7 mV),^14^ but we were not able to show that overexpression of SMIT1 alone directly affected the current amplitude of heteromeric Kv7.4/Kv7.5 channels. The discrepancy between patch-clamp data from VSMCs and CHO cells may reflect a different molecular architecture of the Kv7.4/Kv7.5 channels in the CHO cells compared with arterial smooth muscle cells. In particular, the ancillary subunit KCNE4, which our PLA experiments show interacts with SMIT1 in VSMCs, coassembles with Kv7 channels and alters their function.^35^ Because KCNE subunits modulate the binding of SMIT1 to Kv7.2,^14^ the possibility exists that the lack of effect of SMIT1 overexpression in CHO cells was due to the absence of KCNE4 subunits that could modify SMIT1 interaction with the Kv7.4/Kv7.5 channels. Alternatively, the relative increase in SMIT1 with respect to Kv7 channels in CHO cells is likely to be very different than the situation in smooth muscle cells following raffinose-induced SMIT1 increase. Moreover, we cannot rule out the possibility that additional proteins known to interact with Kv7 channels^12^ might mediate the functional consequences exerted by SMIT1 on Kv7 channels in arteries. Regardless, modulation of SMIT1 protein levels has a marked impact on renal artery reactivity mediated through an increase in Kv7.4/7.5 activity.

Overexpression of SMIT1 potentiates Kv7.1, Kv7.2, and Kv7.3 channels activity by a physical interaction with the channel pore that induces a conformational change affecting the gating and pharmacological properties of Kv7 subunits in the absence of myo-inositol^14^ and via a PIP_2_-dependent mechanism that requires extracellular inositol.^16,17^ Our data suggest that both the physical interaction of SMIT1 and Kv7 channels and localized increase in PIP_2_ via enhanced myo-inositol transport play a role in the regulation of Kv7.4/Kv7.5 channels by SMIT1 in the vasculature. Thus, phlorizin, which prevents SMIT1 transport function and wortmannin, that depletes PIP_2_, only partially prevented the effects on contractility exerted by the incubation with raffinose plus myo-inositol. Furthermore, raffinose alone altered arterial contractility. Synergistic interplay of PIP_2_ with other regulators of Kv7 channels, such as G-protein βγ subunits, have been previously shown to potentiate Kv7.4 currents^44^; therefore, the possibility exists that the interaction of SMIT1 with Kv7.4/Kv7.5 induces a conformational change of the channel that facilitates the binding of PIP_2_.

### SMIT1 Resides in Close Proximity to Kv7.4 and Kv7.5

Physical interaction of SMIT1 with Kv7.1, Kv7.2, and Kv7.3 have been shown in overexpression systems, neurons, and choroid plexus epithelium, where they reciprocally regulate their function.^8,16,17^ Our data showed that in VSMCs SMIT1 resided in close proximity (<40 nm) with both Kv7.4 and Kv7.5 subunits, as well as the accessory subunit KCNE4. Interestingly, PLA also showed that SMIT1 overexpression selectively increased the interaction between SMIT1 and Kv7.4/Kv7.5, without affecting the interaction of these proteins with other molecular partners, such as Kv2.1 or TRPC1. In particular, our PLA data show a preferential interaction of SMIT1 with heteromeric Kv7.4/Kv7.5 channels because knockdown of either Kv7.4 or Kv7.5 reduced the interaction of SMIT1 with both Kv7 subunits, consistent with the reduction of Kv7.4–Kv7.5 heteromers induced by Kv7 subunits silencing. The preferential interaction of SMIT1 with the heteromeric channels is also suggested by PLA data from overexpression system showing that SMIT1-Kv7.4 interaction occurred only when both Kv7 subunits were expressed in CHO cells. These data are consistent with previous evidence showing that SMIT1 neither coimmunoprecipitated with Kv7.4 nor affected the currents mediated by Kv7.4 homomeric channels.^8^ Assembly of heteromeric Kv7 channels occurs very early during protein trafficking to the plasma membrane and is responsible for increased channel insertion and enhanced Kv7-mediated currents.^45,46^ PLA data in overexpression system also suggest that the molecular determinants responsible for this interaction are localized within Kv7.5 because only the Kv7.5 subunit was able to interact with SMIT1 in homomeric conformation, although the possibility exists that SMIT1 binds a complex region resulting from the heteromeric assembly of Kv7.4 and Kv7.5 subunits. Interestingly, interaction of Kv7.5 with SMIT1 seemed to be reduced when Kv7.4 and Kv7.5 were coexpressed in CHO cells. A recent study identified the pore region of Kv7.2 as necessary and sufficient for SMIT1 binding to this subunit. However, the authors suggested that the docking site for SMIT1 on Kv7.2 was large enough to allow further binding of the ancillary KCNE subunits which could, in turn, modulate SMIT1 interaction.^14^ Therefore, the possibility exists that a complex interaction between Kv7.4, Kv7.5, and KCNE4 might occur in VSMCs, possibly increasing the interaction of SMIT1 with Kv7.4/Kv7.5 in native cells. Further studies are needed to identify the molecular determinants of SMIT1 binding on vascular Kv7 channels and clarify the stoichiometry of this interaction. Irrespective of these issues, these data are, to our knowledge, the first evidence of the interaction of an organic osmolyte transporter and a potassium channel in the vasculature.

### Conclusions

Crosstalk between members of the transporters- and ion channels families has been unveiled recently, highlighting a high degree of functional and physical interaction that has led to the creation of the term chansporter.^15^ Here, we describe for the first time a functional and molecular interaction between a myo-inositol transporter and a potassium channels in the vasculature that affects arterial contractility. Such interaction might provide an explanation for the changes in vascular reactivity observed in hyperosmolarity, a condition occurring in pathologies, such as diabetes mellitus.

In conclusion, our data unveil a novel mechanism regulating arterial reactivity and highlight a new potential strategy in the treatment of vascular disease.

## Acknowledgments

We acknowledge the Biological Research Facility for animal services, and the Image Resource Facility for assistance with confocal imaging, both at St George’s University of London. We thank Professor Geoffrey W. Abbott (University of California, Irvine, United States) for the SMIT1 (sodium:myo-inositol transporter 1) plasmid and scientific discussions.

## Sources of Funding

J.B. Stott was funded by a British Heart Foundation grant (PG/15/97/31862) awarded to I.A. Greenwood. S.N. Baldwin was funded by a British Heart Foundation grant (FS/18/41/33762) awarded to I.A. Greenwood. G. Mondejar-Parreño was funded by Ciber Enfermedades Respiratorias, CIBERES, grant (New markets and Therapeutic Targets for the Diagnosis and Treatment of Pulmonary Hypertension, EMPATHY project).

## Disclosures

None.

## Supplementary Material


