# Supplementary material for: SMIT (Sodium-Myo-Inositol Transporter) 1 Regulates Arterial Contractility Through the Modulation of Vascular Kv7 Channels
Source: Arterioscler Thromb Vasc Biol. 2020 Aug 13;40(10):2468–80. doi: 10.1161/ATVBAHA.120.315096 (PMC7505149; doi:10.1161/ATVBAHA.120.315096)
Supplement: Supplementary file 1 [file atv-40-2468-s001.pdf]

SUPPLEMENTAL MATERIALS

The sodium-myo-inositol transporter SMIT1 regulates arterial contractility through the modulation of vascular Kv7 channels

Vincenzo Barrese, MD, PhD[1,2], Jennifer B Stott, BSc (Hons) [1], PhD, Samuel N Baldwin, BSc (Hons)[1], Gema Mondejar-Parreño, BSc (Hons) [3], Iain A Greenwood, BSc (Hons), PhD[1]

[1]Vascular Research Centre, Institute of Molecular & Clinical Sciences, St George's, University of London, London, UK

[2]Department of Neuroscience, Reproductive Sciences and Dentistry, University of Naples Federico II, Naples, Italy

[3]Departament of Pharmacology and Toxicology. School of Medicine, Universidad Complutense de Madrid, Madrid, Spain.

DETAILED MATERIALS AND METHODS

Animals

All experiments were performed in accordance with the UK Animals (Scientific Procedures) Act (1986) and the Spanish Royal Decree 1201/2005 and 53/2013 (Care and Use of Laboratory Animals), and were approved by local Animal Welfare committees (St George's University and Universidad Complutense de Madrid). Gonadal hormones regulate arterial contractility by modulating the response to vasocontrinctors and altering the response. In order to reduce variability due to the effects of sex hormones on arterial contractility, only male rats were used in the present study. 10-12 weeks-old male Wistar rats (Charles River, UK) were killed by cervical dislocation. Animals were housed in a climatically controlled environment, on a 12h light/dark cycle, with free access to water and standard food *ad libitum*.

Arteries

Main conduit renal and third order mesenteric arteries were microdissected and cleaned of adherent fat in ice cold Krebs solution as previously described [1]. Arteries were incubated in a 24-well plate containing DMEM/F-12 culture medium (Sigma Aldrich, Dorset, UK) supplemented with 1% penicillin-streptomycin (Sigma Aldrich, Dorset, UK) in a 37°C incubator with 5% CO_2 for 16 hours (for myo-inositol/raffinose treatments) or 48 hours (for gene-silencing experiments). In double knockdown experiments, arteries were incubated in the morpholinos transfection mix for 32 hours and then incubated in medium containing either vehicle or raffinose plus myo-inositol for 16 hours.

Cell Culture

Chinese Hamster Ovary (CHO) cells were maintained in DMEM supplemented with 10% (v/v) foetal bovine serum, 2 mmol/L L-glutamine, and 1% (v/v) penicillin/streptomycin (Sigma Aldrich, Dorset, UK) in a 37°C incubator with 5% CO_2. CHO cells were plated in 6-well dish and transfected with pcDNA3.1-KCNQ4, pcDNA3.1-KCNQ5, and pcDNA3.1-SMIT1 plasmids using Lipofectamine 2000 (Thermo Fisher, Paisley, UK), according to the manufacturer's instructions. A plasmid encoding for green fluorescent protein (pmaxCloningTM Vector, Lonza, Basel, Switzerland) was also used to determine transfection efficiency and equalise DNA amount for transfections. CHO cells were used 24 hours after transfection.

Isolation of vascular smooth muscle cells

VSMCs were isolated as previously described[1]. Briefly, renal and mesenteric arteries from male Wistar rats were digested with collagenase type IA (2 mg/ml) and protease type X (1 mg/ml); single cells were isolated by gentle mechanical agitation with a Pasteur pipette, and plated on 25 mm diameter coverslips. Cells were allowed to settle for 1 hour at room temperature (RT, 20-22°C) and then used for immunofluorescence experiments. For qPCR experiments, VSMCs were prepared to ensure removal of endothelium as previously described[2]. Briefly, segments of renal artery were longitudinally cut and the lumen was rubbed with a human hair. The arteries were then placed in PBS containing 0.1% Triton X for 1 min followed by three 1-min washes in fresh PBS. The treated arteries were subsequently dispersed to a cell suspension, spun down into a pellet and used for RNA extraction.

Gene knock-down

Knockdown of Kv7.4, Kv7.5 and SMIT1 in renal and mesenteric arteries was performed by transfection with morpholino oligonucleotides as described in vessels previously[3]. Targeting and scrambled (control) morpholino oligonucleotides (5 µmol/L; Genetools, Oregon, USA) were mixed in Opti-MEM and transfected using Lipofectamine 2000 (Thermo Fisher, Paisley, UK). Double knockdown of Kv7.4 and Kv7.5 was obtained by incubating both targeting morpholinos in the same mix (5 µmol/L each); control arteries for these experiments were transfected with 10 µmol/L scrambled morpholino. The mix was then added to the arteries incubated with DMEM/ F-12 with 1% penicillin/streptomycin.

RNA extraction, reverse transcription and quantitative PCR

Total RNA was extracted from arteries using Monarch Total RNA Miniprep Kit (New England Biolabs, Hitchin, UK), according to the manufacturer's instruction, and reverse transcribed to cDNA using Luna Script RT Super Mix ((New England Biolabs, Hitchin, UK). Q-PCR experiments were run in CFX96 Real-Time PCR Detection System (Bio-Rad, Hertfordshire, UK) using the SYBR-Green detection technique and specific primers (see table 1), as previously described [1]. Cycle threshold (Ct) for each gene of interest was calculated using Bio-Rad CFX96 Manager 3.0 software, normalized to two reference genes, namely Ubiqutin C (UBC) and DNA Topoisomerase I (TOP1). Data were expressed using the $2^{-\Delta Ct}$ formula. No template controls and reverse transcriptase-negative controls were run alongside all reactions to assess contamination. Mastermix for the qPCR experiments were purchased from Primerdesign (Southampton, UK). For further information regarding primers, see Supplemental Table I.

Immunofluorescence

VSMCs were fixed with 3% paraformaldehyde (PFA) in PBS solution at RT for 10 min, treated with 0.1 mol/L glycine for 5 min and incubated in blocking solution (PBS containing 0.1% Triton X-100 and 1% bovine serum albumin) for 1 hour at RT. Cells were then incubated overnight at 4°C with a mouse anti-SMIT1 antibody (dilution 1:100, Santa Cruz, Texas, USA) and a rabbit anti-Kv7.4 (dilution 1:200, Abcam, Cambridge, UK) that has been validated in previous studies[4] . Samples were then washed with PBS and incubated for 1 hour with donkey anti-mouse and anti-rabbit secondary antibodies conjugated to Alexa Fluor 488 and Alexa Fluor 567, respectively (dilution 1:100, Thermo-Fisher, Paisley, UK). All antibodies were diluted in blocking solution. Coverslips were washed with PBS and analysed with a Nikon A1R confocal microscope (Nikon Instruments Europe BV, Amsterdam, Netherlands). Corrected total cell fluorescence (CTCF) was calculated using ImageJ software as elsewhere described[5]. The number of cells analysed is indicated by 'n', whereas 'N' represents the number of animals used.

Patch-clamp recordings

All current recordings were made with an Axopatch 200B and a Digidata 1322A (Axon Instruments, Burlingame, CA, USA). For renal VSMCs, membrane Kv currents were measured using perforated whole-cell voltage clamp by including amphotericin B in the pipette solution (final concentration of 200 µg/ml) at room temperature (21-23ºC). Single renal artery myocytes were enzymatically isolated, as described previously[6]. Freshly isolated VSMCs were superfused with a bath solution containing (in mmol/L): 120 NaCl, 3 NaHCO3, 4.2 KCl, 1.2 KH2PO4, 0.5 MgCl2, 1.8 $CaCl_2$, 10

glucose, and 10 HEPES (pH 7.4) and an internal pipette solution contained (in mmol/L): 110 potassium gluconate, 30 KCl, 0.5 MgCl2, 5 HEPES and 0.1 EGTA (pH 7.2). Kv currents were evoked by the application of 325 ms step pulses to between −90 mV to +50 mV in increments of 10 mV from a holding potential of −70 mV. In order to inhibit Kv1, Kv2, Ca^{2+}-activated K^+ currents and voltage-dependent inward Ca^{2+} currents, 4-AP (100 µmol/L), TEA+ (4 mmol/L) and nifedipine (100 nmol/L) were included in the bath solution[6]. The magnitude of the Kv7 component was considered to be the residual current that was sensitive to 10 µmol/L XE991. Current-voltage relationships were constructed by measuring the currents at the end of the pulse. Cell capacitance was calculated from the integral of the capacitive transient current elicited by 10 mV hyperpolarizing pulses from a holding potential of −70 mV. Currents were normalized for cell capacitance and expressed as pA pF–1.

For patch-clamp recording in CHO cells, we used an external solution containing (in mmol/L): 140 NaCl, 4 KCl, 2 CaCl, 1 $MgCl_2$ and 10 HEPES. Patch pipettes with a resistance of 4-12 MΩ were filled with a pipette solution containing (in mmol/L): 110 K gluconate, 30 KCl, 0.5 $MgCl_2$, 5 HEPES and 0.5 EGTA. Cells were held at -60 mV and currents amplitude was monitored by application of a test pulse to 40 mV every 20 s. To generate current-voltage relationships cells were stepped from a holding potential of -60 mV to voltages from -70 to +40 mV for 500 ms at 15 s intervals.

Wire Myography

Segments of renal arteries (~2 mm) were mounted in a wire myograph (Danish Myo Technology, Aarhus, Denmark) for isometric tension recording and kept in physiological saline solution (PSS) containing (in mmol/L): 4.5 KCl, 120 NaCl, 1.2 $MgSO_4.7H_2O$, 1.2 $NaH_2PO_4.2H_2O$, 25 $NaHCO_3$ 5 D-Glucose and 1.25 $CaCl_2$. Arteries were incubated in the myograph chamber with 95% O_2/5% CO_2 at 37°C. Vessels were normalised to 90% of the diameter at 100 mm Hg and challenged with 60 mmol/L KCl to assess viability. Concentration-effect curves for methoxamine (30 nmol/L-30 µmol/L) were constructed to evaluate arterial contractility after the treatments with myo-inositol, raffinose, and morpholino oligonucleotides. In the experiments with the activators of K^+ channels, arteries were contracted twice with methoxamine (10 µmol/L), incubated for 10 minutes with the drug, and then exposed to methoxamine (Fig 3A). Data were recorded and analysed using LabChart® 7 (ADInstruments, Dunedin, New Zealand).

Proximity Ligation Assay

Proximity Ligation Assay (PLA) was used to assess protein-protein interactions as previously described using validated antibodies[1,3]. VSMCs were fixed with 3% PFA for 10 min and permeabilised in PBS-0.1 % Triton X-100 for 5 minutes. The Duolink in situ PLA detection kit (Sigma Aldrich, Dorset, UK) was used according to the manufacturer's protocol. Cells were blocked for 30 min at 37°C in Duolink blocking solution and incubated overnight at 4°C with a combination of two of the following primary antibodies: i) mouse anti-SMIT1 (1:100, Santa Cruz, Texas, USA); ii) rabbit anti-Kv7.4 (1:200, Abcam, Cambridge, UK); iii) rabbit anti Kv7.5 (1:200, Sigma Aldrich, UK); iv) goat anti-Kv7.4 (1:100, Santa Cruz, Texas, USA); v) rabbit anti-KCNE4 (1:200, Sigma Aldrich, UK); v) rabbit anti-Kv2.1 (1:100, Alomone Labs, Jerusalem, Israel); vi) mouse anti-TRPC1 (1:100, Santa Cruz, Texas, USA). Cells were labelled with Duolink anti-rabbit PLUS and anti-mouse or anti-goat MINUS

probes for 1 hour at 37°C. Hybridised oligonucleotides attached to the probes were ligated for 30 mins at 37°C prior to rolling circle amplification for 100 mins at 37°C. Z-stack images were acquired using a Nikon confocal microscope (Nikon Instruments Europe BV, Amsterdam, Netherlands). Analysis of mid-cell xy sections were performed using Image-J software. For PLA in CHO cells, the number of puncta per cell was calculated by dividing the total number of dots by the number of nuclei in the microscopic field.

Drugs

Specific activators of different potassium channels were used at sub-maximal concentrations derived from previous work. These were the Kv7.2-7.5 activators ML213 and retigabine[7] (Tocris Bioscence, Bristol, UK), the Kv7.1 activator RL-3[8] (Sigma Aldrich, Dorset, UK), the K_{ATP} activator levcromakalim[9] (Tocris Bioscence, Bristol, UK) and the BK_{Ca} activator NS11021[10] (Tocris Bioscence, Bristol, UK). Specific blockers of Kv7 channels (XE-991[1], linopirdine and HMR1556[11]), SMIT1 (phlorizin[12]), and the G-Protein-gated Inwardly-Rectifying K+ Channels (Tertiapin Q[13]) were purchased from Tocris Bioscence, Bristol, UK. Hypertonic treatment by incubation in a media containing 150 mmol/L raffinose along with 1 mmol/L myo-inositol was used to increase SMIT1 abundance and activity based upon previous work[14]. The inhibitor of phosphatidylinositol 3-kinase and phosphatidylinositol 4-kinase wortmannin was purchased from Sigma Aldrich, Dorset, UK.

Statistical analysis

All data are expressed as mean ± S.E.M. One- or two-way ANOVA test followed by a Dunnett’s or Tukey’s multiple comparisons test, and Student’s t-test (paired or unpaired) were used to determine statistical significance between groups, according to the different experiments. Normality and variance of the data were not tested. The significance level for statistic tests was 0.05 (differences were considered statistically significant when $p<0.05$). Asterisks in the figures indicate the approximate p value given by the test, as described in the figure legends.

SUPPLEMENTAL TABLE I. List of primers used for qPCR

Gene	(+) Forward primer sequence	(-) Reverse primer sequence	Gene accession number	Amplicon (bp)
Kcnq4	GAATGAGCAGCTC CCAGAAG	AAGCTCCAGCTTT TCTGCAC	XM_233477	133
Kcnq5	AACTGATGAGGAG GTCGGTG	GATGACCGTGACC TTCCAGT	XM_001071249.3	120
Slc5a11	TCCCTGGTATGGT GAGCAGA	CTGTGGGCAAAAG TTCCAGC	NM_001100482.2	141
Scla3	GCATTAAGGGGCT CCAACCT	ATGAGAGCTGCTT GCCCATT	NM_053715.2	136

TOP1 and UBC were used from PrimerDesign geNorm Reference Gene Selection Kit and were prevalidated. For proprietary reasons no information can be given for the reference genes.

SUPPLEMENTAL FIGURES

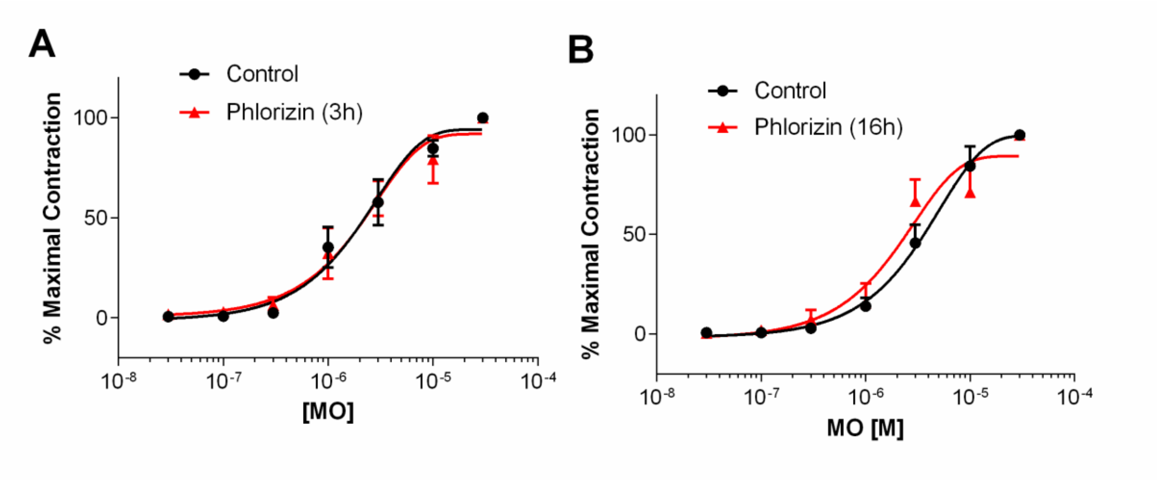

Supplemental Figure I. Effects of phlorizin on renal artery contractility. Isometric tension recordings showing the effects of increasing concentration of methoxamine (MO) in segments of renal arteries after incubation for 3 hours (A) or 3h (A) or 16 hours (B) with vehicle (control, black line), or 500 µmol/L phlorizin (red line). Data are shown as percentage of the contraction obtained with 30 µmol/L methoxamine. Data are expressed as mean±S.E.M. N=6 per experimental group.

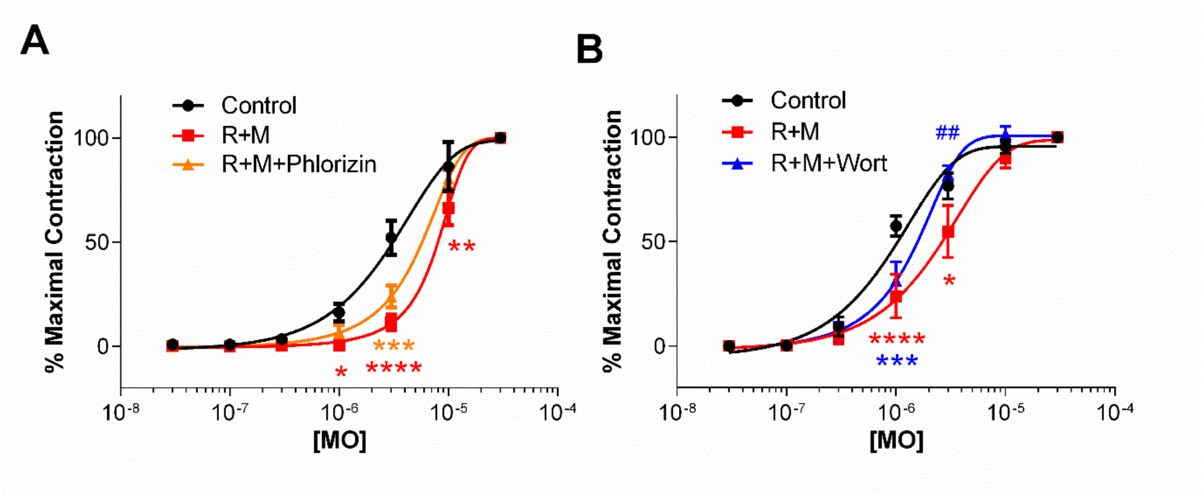

Supplemental Figure II. Effects of phlorizin and wortmannin on enhanced SMIT1 expression. Isometric tension recordings showing the effects of increasing concentration of methoxamine (MO) in the presence of 500 µmol/L phlorizin (A) or 10 nmol/L wortmannin (B) in segments of renal arteries after 16 hours incubation with vehicle or 150 mmol/L raffinose plus 1 mmol/L myo-inositol. Data are shown as percentage of the contraction obtained with 30 µmol/L methoxamine. Data are expressed as mean±S.E.M. N=6 per experimental group. *=p<0.05; **=p<0.01; ***=p<0.001: ****=p<0.0001 vs Control; ##=p<0.01 vs R+M (red lines); N=6 for each experimental point.

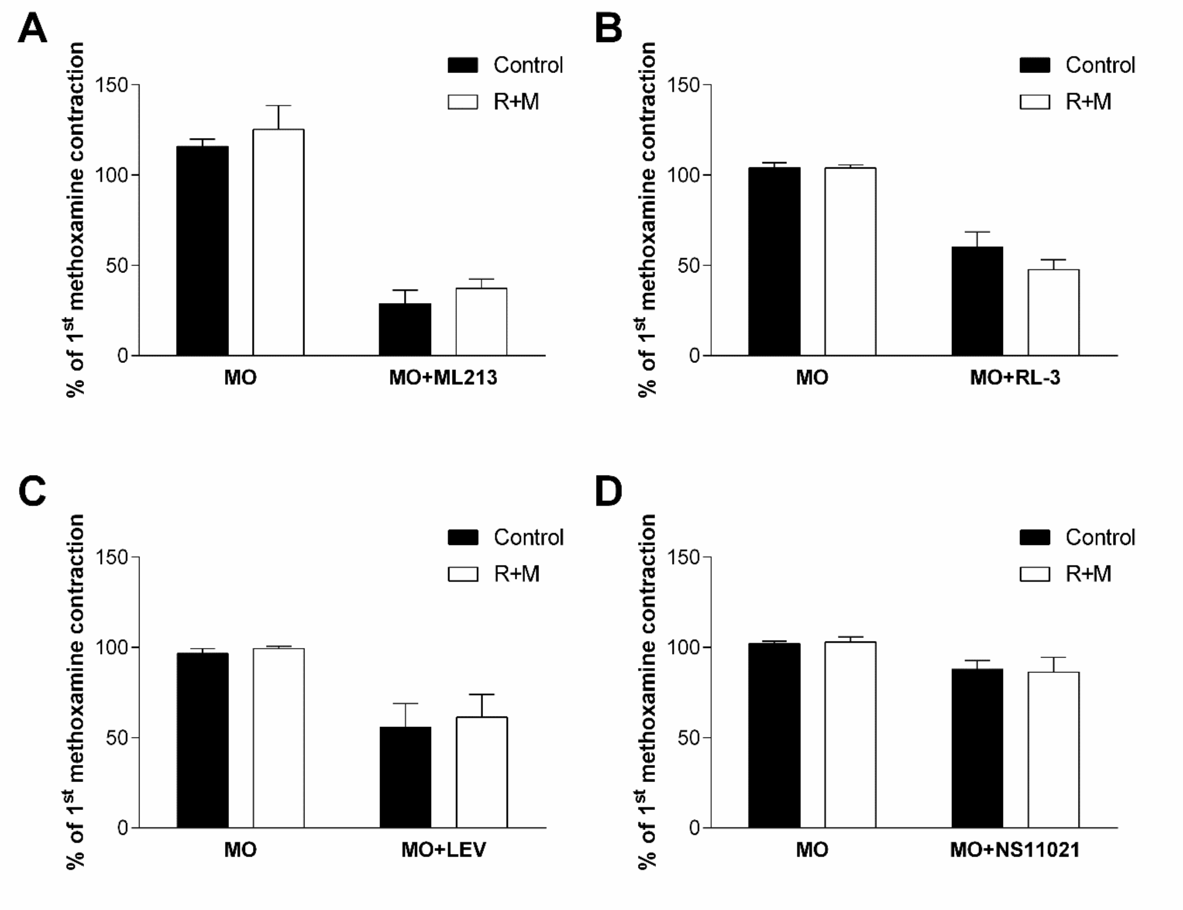

Supplemental Figure III. Effects of enhanced SMIT1 expression on K$^+$ channels-activators. Quantification of the responses to methoxamine in the presence of ML213 (A, N=9), RL-3 (B, N=6), levcromakalim (C, N=8), or NS11021 (D, N=4) in renal arteries incubated with vehicle (Control, black bars) or raffinose plus myo-inositol (R+M, white bars). Data are expressed as mean±S.E.M. and shown as percentage of the contraction to the first stimulation with methoxamine (see Fig 3A). The responses to the second stimulation with methoxamine before the incubation with the activators (MO) are also shown.

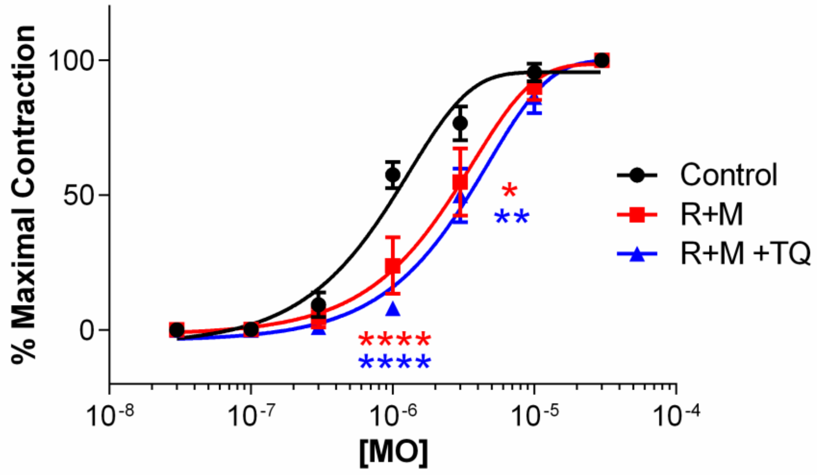

Supplemental Figure IV. Effects of GIRK channels block on enhanced SMIT1 expression. Isometric tension recordings showing the effects of increasing concentration of methoxamine (MO) in the presence of (1 µmol/L tertiapin Q in segments of renal arteries after 16 hours incubation with vehicle or 150 mmol/L raffinose plus 1 mmol/L myo-inositol. Data are shown as percentage of the contraction obtained with 30 µmol/L methoxamine. Data are expressed as mean±S.E.M. N=6 per experimental group. *=p<0.05; **=p<0.01; ****=p<0.0001 vs Control.

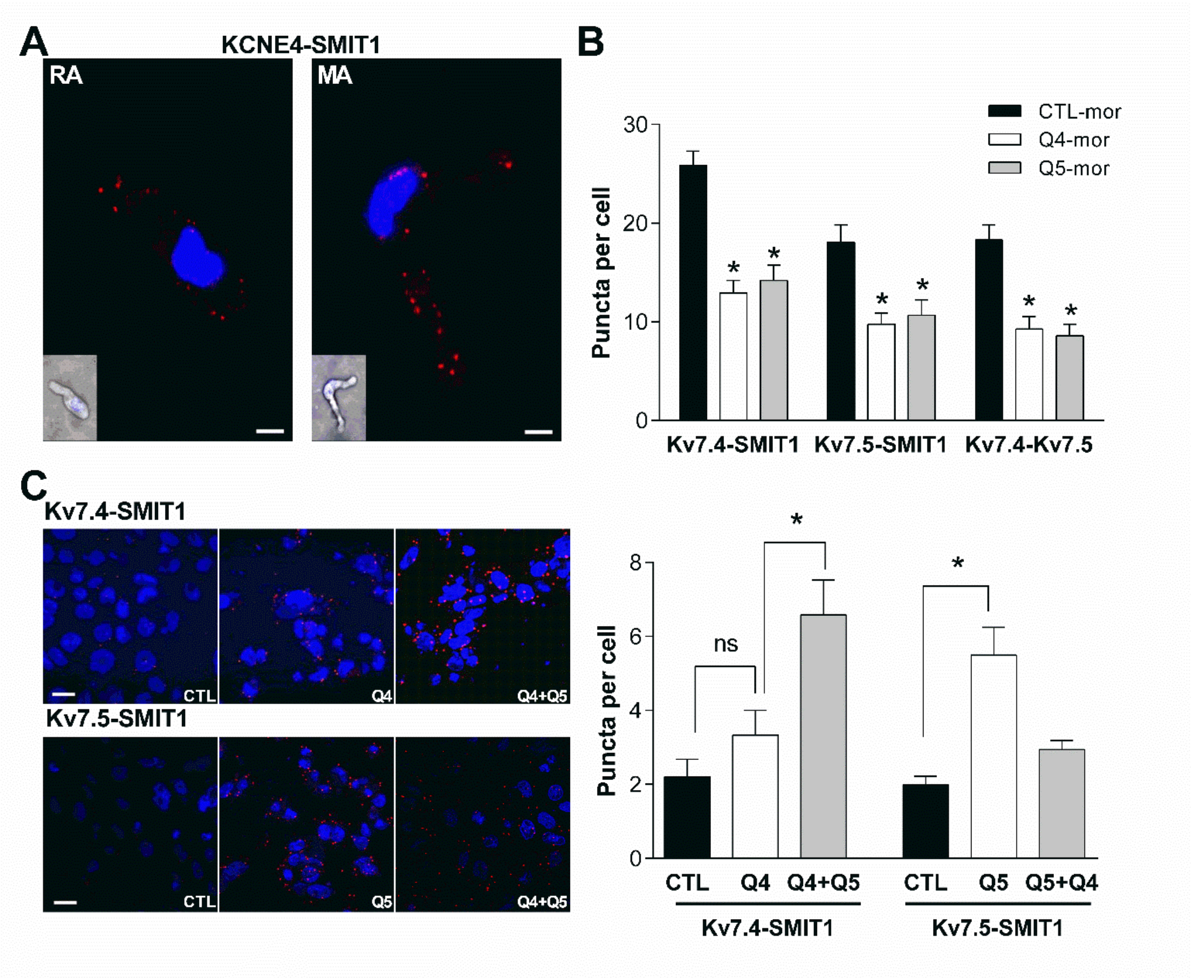

Supplemental Figure V. Interaction of SMIT1 KCNE4 subunit Kv7.4/Kv7.5 channels. (A) Proximity Ligation Assay (PLA) showing the interaction of SMIT1 with KCNE4 in VSMCs isolated from renal (RA) and mesenteric (MA) arteries. The insets show a brightfield image of the cell. Nuclei (DAPI staining, blue) are also shown. Scale bar=5 µm. (B) Quantification of Proximity Ligation Assays (PLA) showing the interaction between Kv7.4-SMIT1, Kv7.5-SMIT1, and Kv7.4-Kv7.5 in mesenteric VSMCs. Arteries were incubated with a scrambled morpholino (CTL-mor, black bars), a morpholino targeting Kv7.4 (Q4-mor, white bars) or Kv7.5 (Q5-mor, grey bars). Data represent the mean number of PLA signals per mid-cell xy section, expressed as mean±S.E.M. n=26-36 cells from 3-4 rats (N=3-4) per experimental point in 3-4 sessions. *=p<0.05 vs respective controls (CTL-mor). (C) PLA showing the interaction of SMIT1 with Kv7.4 and Kv7.5 in CHO cells transfected with different combination of plasmids encoding for green fluorescent protein (pMAX, control), Kv7.4 (Q4) and Kv7.5 (Q5), as indicated. Nuclei (DAPI staining, blue) are also shown. Scale bar=10 µm. (C) Bar graphs showing the quantification of the mean number of PLA signals per mid-cells xy section in transfected CHO cells. Data are expressed as mean±S.E.M.; n=20-30 microscopic fields from 4-6 batches of cells per experimental point in 4-6 sessions. *=p<0.05, ns= not significant.

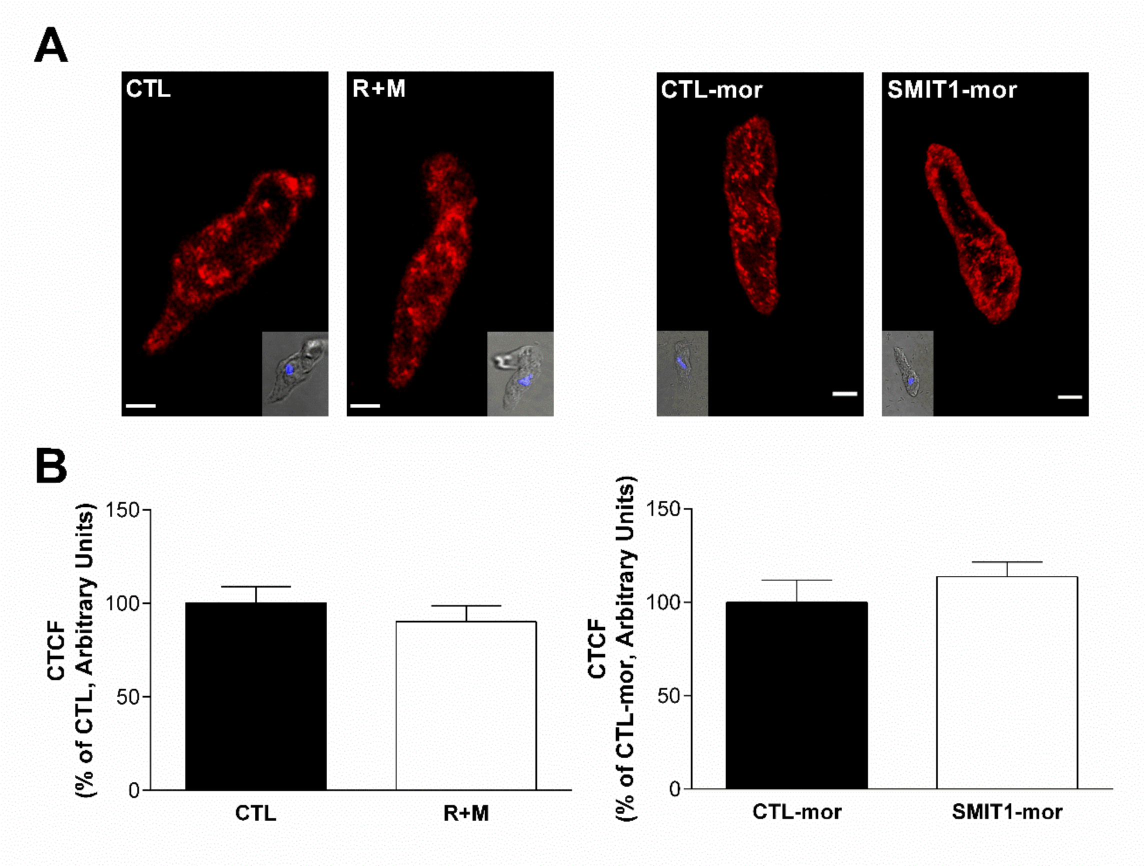

Supplemental Figure VI. Effects of the modulation of SMIT1 levels on Kv7.4 expression in renal VSMCs. (A) Representative images showing the immunostaining of Kv7.4 in renal VSMCs incubated with vehicle (CTL) or raffinose plus myo-inositol (R+M) (leftmost panels), scrambled morpholino (CTL-mor) or a morpholino targeting SMIT1 (SMIT1-mor) (rightmost panels). The insets show brightfield images of the cells. Scale bar=5 µm. (B) bar graphs showing the quantification of Kv7.4 fluorescence intensities in VSMCs incubated with R+M (left) or SMIT1-mor (right), with their respective controls. Fluorescence intensities are measured as corrected total cell fluorescence (CTCF) and expressed as percentage of control (vehicle-incubated arteries or scrambled morpholino). Data are expressed as mean±S.E.M. n=35-40 cells from 4 different preparations (N=4) per experimental group.

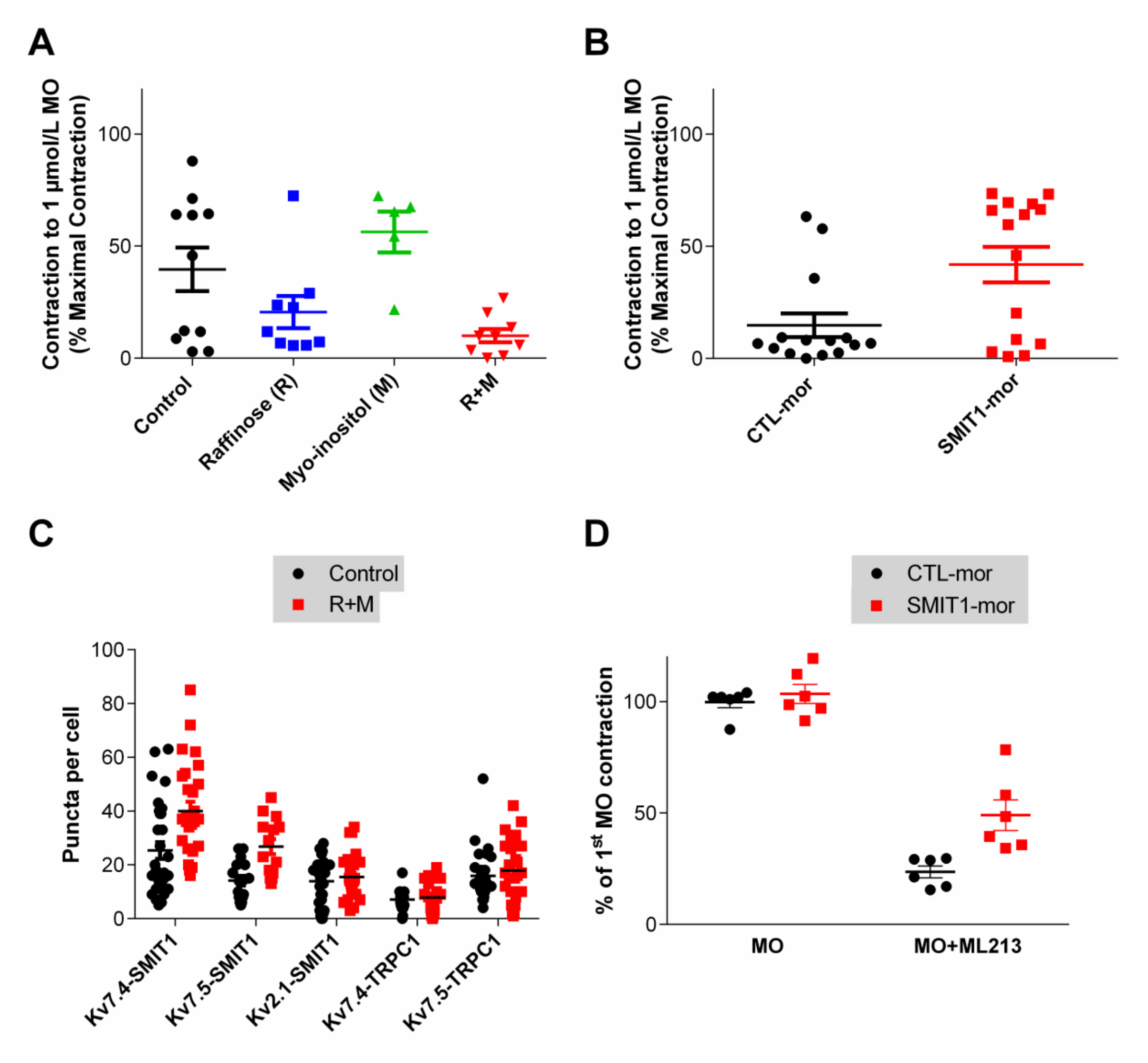

Supplemental Figure VII. Scatter dot plot for myography and PLA experiments. Scatter dot plot showing the distribution of values for: (A) contraction to 1 μmol/L methoxamine (MO) in renal arteries incubated with vehicle (control), 150 mmol/L raffinose, 1 mmol/L myo-inositol, or 150 mmol/L raffinose plus 1 mmol/L myo-inositol (R+M) (see also Fig 2B); (B) contraction to 1 μmol/L MO in renal arteries incubated with a scrambled morpholino (CTL-mor) or a morpholino targeting SMIT1 (SMIT1-mor) (see also Fig 2A). Data are shown as percentage of the contraction to 30 μmol/L MO. (C) PLA puncta for the indicated couples of antibodies in arteries incubated in vehicle (control) or 150 mmol/L raffinose plus 1 mmol/L myo-inositol (R+M) (see also Fig 6B). (D) responses to 10 μmol/L MO in the presence of ML213 in renal arteries incubated with a scrambled (CTL-mor) or an anti-SMIT1 morpholino oligonucleotide (SMIT1-mor). Data are shown as percentage of the contraction to the first stimulation with MO (see also Fig 3A-B). The responses to the second incubation of methoxamine before the incubation with ML213 (MO) are also shown.

REFERENCES

1. Barrese V, Stott JB, Figueiredo HB, Aubdool AA, Hobbs AJ, Jepps TA, McNeish AJ, Greenwood IA. Angiotensin II Promotes KV7.4 Channels Degradation Through Reduced Interaction With HSP90 (Heat Shock Protein 90). *Hypertens Dallas Tex 1979*. 2018;71:1091–1100.

2. Page HRA, Dalsgaard T, Baldwin SN, Jepps TA, Povstyan O, Olesen SP, Greenwood IA. TMEM16A is implicated in the regulation of coronary flow and is altered in hypertension. *Br J Pharmacol*. 2019;176:1635–1648.

3. Stott JB, Barrese V, Suresh M, Masoodi S, Greenwood IA. Investigating the Role of G Protein βγ in Kv7-Dependent Relaxations of the Rat Vasculature. *Arterioscler Thromb Vasc Biol*. 2018;38:2091–2102.

4. Neverisky DL, Abbott GW. KCNQ-SMIT complex formation facilitates ion channel-solute transporter cross talk. *FASEB J*. 2017;31:2828–2838.

5. Burgess A, Vigneron S, Brioudes E, Labbé J-C, Lorca T, Castro A. Loss of human Greatwall results in G2 arrest and multiple mitotic defects due to deregulation of the cyclin B-Cdc2/PP2A balance. *Proc Natl Acad Sci*. 2010;107:12564–12569.

6. Chadha Preet S., Zunke Friederike, Zhu Hai-Lei, Davis Alison J., Jepps Thomas A., Olesen Søren P., Cole William C., Moffatt James D., Greenwood Iain A. Reduced KCNQ4-Encoded Voltage-Dependent Potassium Channel Activity Underlies Impaired β-Adrenoceptor–Mediated Relaxation of Renal Arteries in Hypertension. *Hypertension*. 2012;59:877–884.

7. Jepps TA, Bentzen BH, Stott JB, Povstyan OV, Sivaloganathan K, Dalby-Brown W, Greenwood IA. Vasorelaxant effects of novel Kv7.4 channel enhancers ML213 and NS15370. *Br J Pharmacol*. 2014;171:4413–4424.

8. Chadha PS, Zunke F, Davis AJ, Jepps TA, Linders JT, Schwake M, Towart R, Greenwood IA. Pharmacological dissection of Kv7.1 channels in systemic and pulmonary arteries. *Br J Pharmacol*. 2012;166:1377–1387.

9. Haba M, Hatakeyama N, Kinoshita H, Teramae H, Azma T, Hatano Y, Matsuda N. The Modulation of Vascular ATP-Sensitive K+ Channel Function via the Phosphatidylinositol 3-Kinase–Akt Pathway Activated by Phenylephrine. *J Pharmacol Exp Ther*. 2010;334:673–678.

10. Shi L, Zhang H, Chen Y, Liu Y, Lu N, Zhao T, Zhang L. Chronic exercise normalizes changes in Cav1.2 and KCa1.1 channels in mesenteric arteries from spontaneously hypertensive rats. *Br J Pharmacol*. 2015;172:1846–1858.

11. Stott JB, Barrese V, Jepps TA, Leighton EV, Greenwood IA. Contribution of Kv7 channels to natriuretic peptide mediated vasodilation in normal and hypertensive rats. *Hypertens Dallas Tex 1979*. 2015;65:676–682.

12. Abbott GW, Tai K-K, Neverisky DL, Hansler A, Hu Z, Roepke TK, Lerner DJ, Chen Q, Liu L, Zupan B, Toth M, Haynes R, Huang X, Demirbas D, Buccafusca R,

Gross SS, Kanda VA, Berry GT. KCNQ1, KCNE2, and Na+-Coupled Solute Transporters Form Reciprocally Regulating Complexes That Affect Neuronal Excitability. *Sci Signal*. 2014;7:ra22–ra22.

13. Li Q, Pang M, Zhu M, Chen L. G-Protein-gated Inwardly-Rectifying K+ Channels and Large-conductance Calcium-Activated K+ Channels Are Involved in C-Type Natriuretic Peptide-Mediated Vasodilation in Human Arteries. *Int Heart J*. 2019;60:168–174.

14. Dai G, Yu H, Kruse M, Traynor-Kaplan A, Hille B. Osmoregulatory inositol transporter SMIT1 modulates electrical activity by adjusting PI(4,5)P $_2$ levels. *Proc Natl Acad Sci*. 2016;113:E3290–E3299.

Major Resources Tables

Animals

Species	Vendor or Source	Background Strain	Sex
Rattus norvegicus	Charles River	Wistar	Male

Antibodies

Target antigen	Vendor or Source	Catalog #	Working concentration
SMIT1	Santa Cruz, Texas, USA	sc-293330	1:100
Kv7.4	Abcam, Cambridge, UK	ab65797	1:100
Kv7.4	Santa Cruz, Texas, USA	sc-20882	1:100
Kv7.5	Sigma Aldrich, UK	ABN-1372	1:200
KCNE4	Sigma Aldrich, UK	HPA011420	1:200
Kv2.1	Alomone Labs, Jerusalem, Israel	APC-012	1:100
TRPC1	Santa Cruz, Texas, USA	sc-133076	1:100

Cultured Cells

Name	Vendor or Source	Sex (F, M, or unknown)
Chinese Hamster Ovary	ATCC (American Type Culture Collection)	F
