# Supplementary figures and images for: SMIT (Sodium-Myo-Inositol Transporter) 1 Regulates Arterial Contractility Through the Modulation of Vascular Kv7 Channels
Source: Arterioscler Thromb Vasc Biol. 2020 Aug 13;40(10):2468–80. doi: 10.1161/ATVBAHA.120.315096 (PMC7505149; doi:10.1161/ATVBAHA.120.315096)

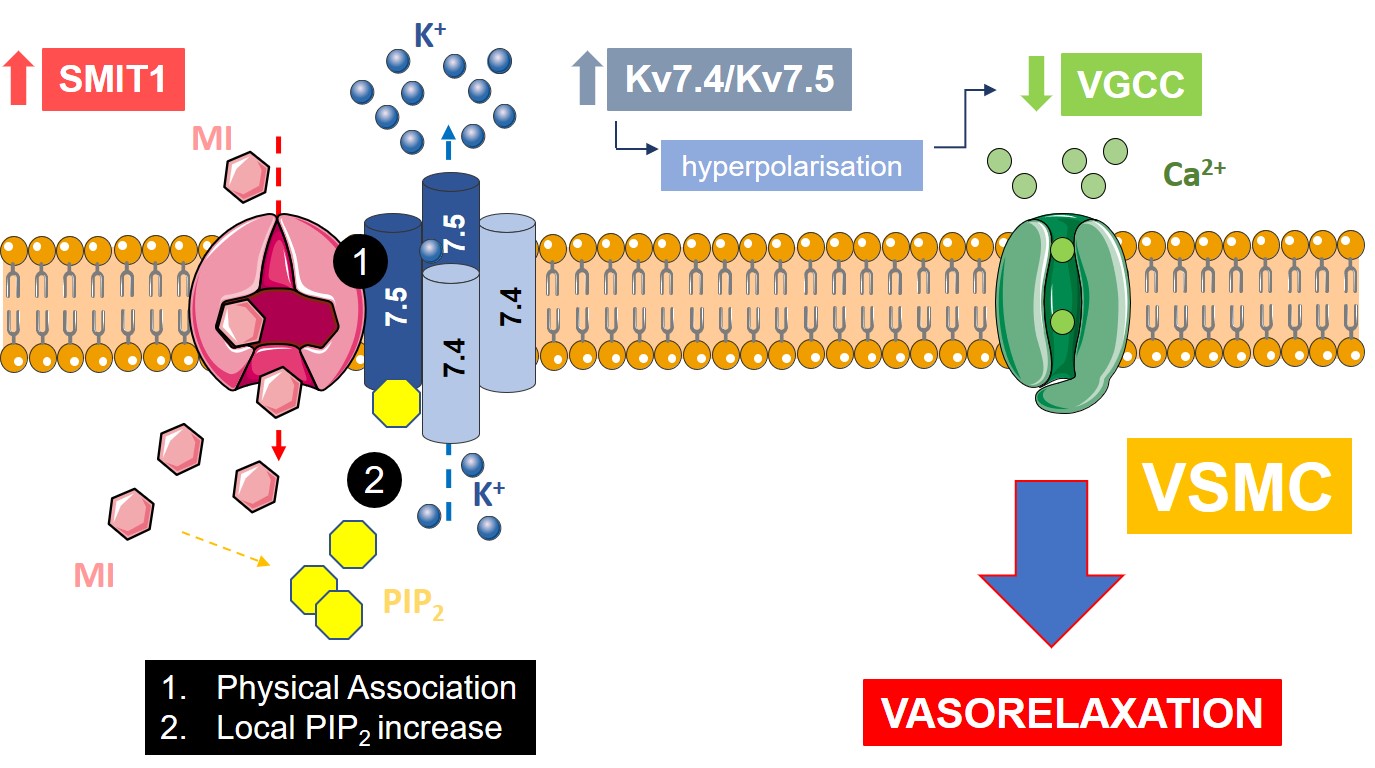

Supplement: Supplementary file 2 [file atv-40-2468-s002.jpg]
